# Mid-Level Data Fusion Techniques of LC-MS and HS-GC-MS for Distinguishing Green and Ripe Forsythiae Fructus

**DOI:** 10.3390/molecules30071404

**Published:** 2025-03-21

**Authors:** Qingling Xie, Hanwen Yuan, Shiqi Liu, Ling Liang, Jiangyi Luo, Mengyun Wang, Bin Li, Wei Wang

**Affiliations:** TCM and Ethnomedicine Innovation & Development International Laboratory, School of Pharmacy, Hunan University of Chinese Medicine, Changsha 410208, China; xieql12@126.com (Q.X.); hanwyuan@hnucm.edu.cn (H.Y.); shiqiliu670@163.com (S.L.); lliang901@stu.hnucm.edu.cn (L.L.); jyiluo1998@163.com (J.L.); 20183362@stu.hnucm.edu.cn (M.W.); libin@hnucm.edu.cn (B.L.)

**Keywords:** Forsythiae Fructus, LC-MS, HS-GC-MS, mid-level data fusion

## Abstract

*Forsythia suspensa* is a crucial plant resource due to its considerable edible and medicinal value. Its fruit, named Forsythiae Fructus (FF), has been widely used in Traditional Chinese Medicine (TCM). According to the fruit maturity stage, FF is categorized into GFF (green Forsythiae Fructus) and RFF (ripe Forsythiae Fructus). In this study, metabolomics based on UPLC-Q/Orbitrap MS and HS-GC-MS, combined with chemometric methods, was employed to differentiate GFF from RFF and identify potential differential metabolites. Additionally, the mid-level data fusion method was employed to integrate data from both techniques, and the performance of the OPLS-DA model (R^2^Y = 0.986, Q^2^ = 0.974) surpassed that of the single HS-GC-MS technique (R^2^Y = 0.968, Q^2^ = 0.930). Moreover, using the criteria of VIP > 1 and *p*-value < 0.05, 30 differential compounds were selected via mid-level data fusion, compared to the initial 61 differential compounds identified by single techniques, effectively reducing data noise and eliminating irrelevant variables. This study provides a comprehensive analysis of volatile and non-volatile compounds in FF, offering valuable insights into quality control and clinical differentiation between GFF and RFF. The findings highlight the potential use of multi-technology metabolomics in the quality control of TCM and offer new perspectives for future research on medicinal plants.

## 1. Introduction

*Forsythia suspensa* is regarded as an important plant due to its high value as both an edible and medicinal resource. It is widely distributed in northern and central China, particularly in Shaanxi, Shanxi, Henan, Shandong, and Anhui, which serve as its major production regions. The different parts of *Forsythia suspensa*, such as flowers, leaves, and fruit, possess significant pharmacological effects [1]. Forsythiae Fructus (FF), also known as Lianqiao in Chinese, is derived from the dried fruit. It has been used in Traditional Chinese Medicine (TCM) for centuries due to its potent heat-clearing, detoxifying, and stasis-dissipating properties [2]. Pharmacological research indicates that FF exhibits properties such as anti-inflammatory, antioxidant, antibacterial, anticancer, antiviral, anti-allergic, and neuroprotective activities [3,4]. The medicinal effects of FF are due to its abundant bioactive compounds, including phenylethanoid glycosides, lignans, terpenoids, flavonoids, and volatile oils [5]. According to Chinese Pharmacopoeia (2020 edition), forsythiaside A and phillyrin are considered major quality evaluation indicators of FF [6]. The complex compound composition of FF makes it insufficient to rely solely on high-performance liquid chromatography (HPLC) based on non-volatile components for quality control, as it fails to comprehensively reflect its overall characteristics and quality [7].

Additionally, the concentration of chemical composition in FF is impacted by its degree of maturity and regional geographical differences. In the TCM system, it is classified into Qingqiao (green Forsythiae Fructus, GFF) and Laoqiao (ripe Forsythiae Fructus, RFF) based on maturity [5]. Although both of them are included as FF in the Chinese Pharmacopoeia, the harvest time may influence their qualitative characteristics and relative content of compounds, thereby further affecting their pharmacological activity [7,8,9]. Therefore, a comprehensive characterization of non-volatile components such as phenylethanoid glycosides and lignans, along with the volatile compounds in essential oils, and the exploration of their differential metabolites, is of significant importance for distinguishing the clinical applications of GFF and RFF.

Metabolomics, as an emerging technology, has proven to be of significant value in the effective discovery of potential differential compounds from TCMs and medicinal plants [10,11,12]. The main analytical platforms used in metabolomics include LC-MS, GC-MS, and NMR [13,14,15,16]. The integration of multiple analytical techniques with chemometric methods provided comprehensive compound characterization, reduced background noise, and highlighted key information, improving classification performance more effectively than single techniques [17,18,19]. However, due to the high-dimensional and complex nature of metabolomics data, multi-technology metabolomics encompasses rich and intricate compositional information, which can easily lead to data redundancy. Therefore, appropriate methods are required for data integration and effective information extraction.

In recent years, data fusion has gained widespread attention for its ability to integrate multi-source data, provide more comprehensive characterization, and reduce errors associated with single data sources through cross-validation and information complementarity [20,21,22]. Data fusion was categorized into three types: low-level, mid-level, and high-level fusion [23]. In low-level data fusion, raw data from various sources were combined without feature selection or processing. Its main drawback was the increase in irrelevant variables, which caused information redundancy. By contrast, mid-level data fusion, which combines the most discriminative variables, significantly enhances classification performance and shows greater discriminative ability [24]. Recent studies have utilized data fusion from different chromatographic and spectroscopic techniques for sample differentiation. For example, Rivera-Pérez et al. successfully integrated ^1^H NMR, UHPLC-HRMS, and GC-HRMS data, improving the classification accuracy of thyme and providing a more comprehensive representation of its chemical profile [14]. Similarly, Gao et al. developed a qualitative discrimination model based on near-infrared (NIR), mid-infrared (MIR), and microscopic Raman spectra, combined with individual spectra and multispectral data fusion strategies, to achieve rapid and effective identification of Hebei yam [25]. These studies demonstrate the potential of multi-technology methods in distinguishing complex samples by integrating information from different data sources and providing a more comprehensive analysis.

So far, the chemical composition analysis of FF mainly focused on single methods, including HPLC, GC-MS, and LC-MS, with the application of multi-technology metabolomics in the classification of GFF and RFF remaining limited. Relying solely on specific compounds for such comparison presents several limitations. First, the chosen compounds might not comprehensively capture the complexity of the sample, as metabolic processes often involve a broad range of compounds, and focusing on a limited number could lead to the omission of essential components. Second, the results are highly dependent on the instrument and method employed, as different analytical instruments, such as GC-MS and LC-MS, possess inherent disparities in sensitivity, resolution, and quantification accuracy, which can impact the detection and measurement of compounds. Therefore, the integration of multiple analytical techniques is crucial to reduce bias and enhance the reliability of results.

This study aims to systematically distinguish GFF and RFF using metabolomics approaches and to identify potential differential metabolites. UPLC-Q/Orbitrap MS and HS-GC-MS were employed to comprehensively analyze the non-volatile and volatile components of FF, combined with chemometric methods to enhance classification accuracy. Furthermore, a mid-level data fusion strategy was implemented to integrate multi-platform data, evaluating its effectiveness in differentiating GFF from RFF and improving data interpretation. Through the identification of differential compounds, this study provides a scientific basis for the quality control and clinical application of FF and further demonstrates the applicability of multi-platform metabolomics in Traditional Chinese Medicine (TCM) research.

## 2. Results and Discussion

### 2.1. Characterization of Non-Volatile Metabolites in GFF and RFF

**Figure 1:**
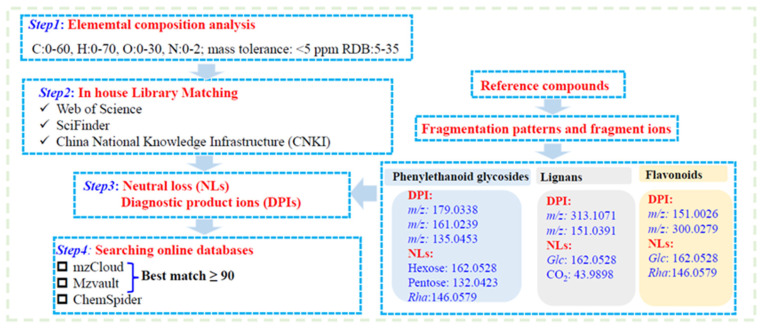
The strategy for identifying and characterizing the chemical constituents of FF.

**Figure 2:**
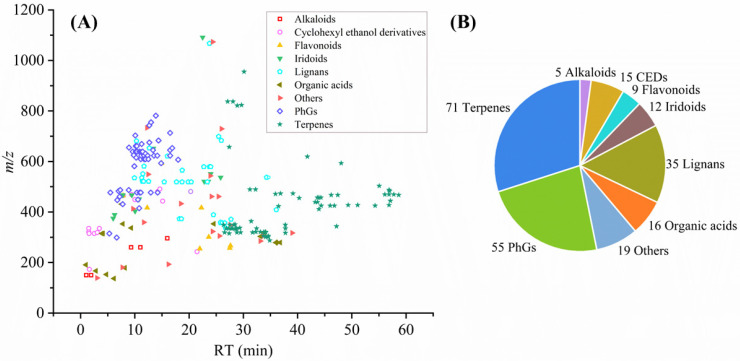
The compounds identified in the QC sample by LC-MS: (**A**) a 2D scatter plot with *m*/*z* versus RT (min); (**B**) a pie chart displaying the number of different types of compounds.

The total ion chromatograms (TIC) for the QC sample, shown in Figure S1, were obtained in both ion modes. The compound identification process was based on high-resolution mass spectrometry (HRMS), which allowed the precise determination of molecular formulas by measuring the exact mass-to-charge ratio (*m*/*z*) of molecular ions. In addition to molecular formula, retention time, and fragmentation patterns, isotopic distribution was also a critical parameter for confirming the structural characteristics of the identified compounds. According to the compound identification strategy (Figure 1), 237 compounds were tentatively identified from the QC samples. Among these, 21 compounds were unambiguously identified by matching retention times and mass spectra with reference compounds. Some of them were excluded due to unavailable MS/MS data. The detailed information of the identified compounds by LC-MS analysis is presented in Table S1. Figure 2 illustrates the distribution and chemical structure types of these compounds identified by LC-MS. Different types of compounds may have similar *m*/*z* or retention times. For example, phenylethanoid glycosides, which have high polarity, are mainly concentrated within a retention time of 0–15 min, whereas terpenoids, with lower polarity, are more widely distributed. The pie chart illustrates the proportion of different compound categories, with terpenoids being the most abundant, followed by phenylethanoid glycosides and lignans.

#### 2.1.1. Identification of Phenylethanoid Glycosides

**Figure 3:**
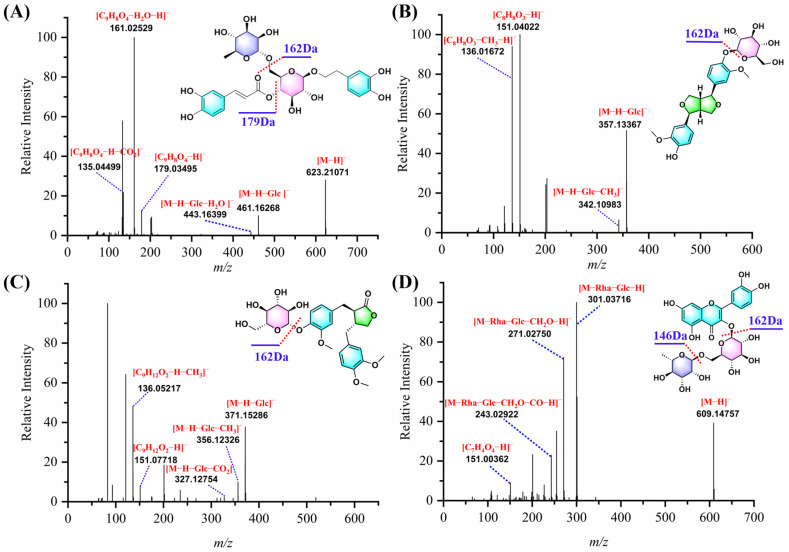
The MS/MS spectra of (**A**) forsythoside A, (**B**) (+)-pinoresinol-4′-*O*-*β*-D-glucopyranoside, (**C**) arctiin, and (**D**) rutin in ESI^−^ mode.

Phenylethanoid glycosides (PhGs) are the active and characteristic compounds in FF, with forsythoside A and forsythoside E being the main representatives [26]. The basic structure of PhGs consists of phenylethyl alcohol and glycosyl moieties. The typical sugar groups include glucose, rhamnose, xylose, and arabinose [27]. These compounds exhibit significant anti-inflammatory, antibacterial, antiviral, and antioxidant properties in both in vitro and in vivo studies [5]. In this study, 56 phenylethanoid glycosides were characterized. Among them, 9 phenylethanoid glycosides (**21**, **24**, **75**, **82**, **90**, **94**, **96**, **98**, and **110**) were confirmed by comparing retention times, [M−H]^−^ ion masses, and MS/MS spectra with reference compounds. In negative mode, these standards were prone to losing sugar moieties, such as rhamnose (146 Da, C_6_H_10_O_4_) and glucose (162 Da, C_6_H_10_O_5_), as well as caffeoyl units (162 Da, C_9_H_6_O_3_), ultimately generating phenylethanoid-related ions at *m*/*z* 153.0557 [C_8_H_10_O_3_−H]^−^ or 137.0608 [C_8_H_10_O_2_−H]^−^ (Figure S2A). Fragment ions at *m*/*z* 461.1627 [M−H−Glc]^−^, 443.1640 [M−H−Glc−H_2_O]^−^, and 315.1085 [M−H−C_9_H_6_O_3_−C_6_H_10_O_4_]^−^ were observed in the MS/MS spectrum of forsythoside A (Figure 3A), attributed to the loss of glucose, H_2_O, and caffeoyl units.

The “Neutral Loss” mode was introduced to quickly screen compounds with common neutral loss groups at *m*/*z* 162, 146, 60, or 44 Da, associated with glucose, rhamnose, acetic acid, and CO_2_, respectively. For example, 21 compounds were successfully screened with a neutral loss of a caffeoyl group (162 Da, C_9_H_6_O_3_). Among them, six compounds (**75**, **90**, **94**, **96**, **98**, and **110**) were characterized as forsythoside I, forsythoside B, forsythoside A, acteoside, calceolarioside B, and isoacteoside, respectively, based on comparisons with reference compounds. Notably, peaks **47**, **49**, **50**, **53**, **78**, **91**, and **109** were identified as potential new compounds since they did not match any entries in the in-house database and online resources (ChemSpider and SciFinder).

Additionally, fragment ions associated with caffeic acid at *m*/*z* 179.0348 [C_9_H_8_O_4_−H]^−^, 161.0245 [C_9_H_8_O_4_−H−H_2_O]^−^, and 135.0422 [C_9_H_8_O_4_−H−CO_2_]^−^ were detected and recognized as Diagnostic Product Ions (DPIs) for compound screening and identification. Moreover, the isomers (**75**, **82**, **93**, **94**, **96**, and **110**) were difficult to identify due to the identical precursor ion at *m*/*z* 623.1981 [M−H]^−^ (calculated for C_29_H_36_O_15_) and similar fragmentation characteristics (Figure S2A). Clog *p* values, which are calculated based on factors such as molecular weight, hydrophobicity, and functional groups, indicate the polarity of the compounds. Higher Clog *p* values indicate lower polarity, resulting in longer retention times in reversed-phase chromatography [28]. In this study, Chemdraw Ultra 14.0 was used to calculate the Clog *p* values of the compounds. Isoacteoside had a higher Clog *p* value (−0.057) than acteoside (−0.890), suggesting its later elution (RT = 16.51 min) compared to acteoside (RT = 14.15 min). Accordingly, the isomers were distinguished.

#### 2.1.2. Identification of Lignans

Lignans in FF can be classified into two types based on their structural skeletons, including furofuran and 2,3-dibenzylbutyrolactone [29]. To facilitate the structural identification and classification of lignan compounds, this study summarized the fragmentation pattern of the two types of lignan using reference standards of (+)-pinoresinol-4′-*O*-*β*-D-glucopyranoside and arctiin as examples. (+)-Pinoresinol-4′-*O*-*β*-D-glucopyranoside presented [M−H]^−^ at *m*/*z* 519.1886 with the molecular formula C_26_H_32_O_11_ (Figure 3B). Its main fragment ions at *m*/*z* 357.1337 [M−H−C_6_H_10_O_5_]^−^, 342.1098 [M−H−C_6_H_10_O_5_−CH_3_]^−^, and 311.1312 [M−H−C_6_H_10_O_5_−CH_3_−CH_2_O]^−^ resulted from the successive loss of glucose (162Da, C_6_H_10_O_5_), CH_3_, and CH_2_O, respectively. Moreover, fragment ions at *m*/*z* 151.0402 [C_8_H_8_O_3_−H]^−^ and 136.0167 [C_8_H_8_O_3_−H−CH_3_]^−^ were also detected. Compounds **105**, **113**, **118**, **120**, **122**, and **129** exhibited the same deprotonated ion [M−H]^−^ at *m*/*z* 535.1794, aligning with the formula C_26_H_32_O_11_. Compound 105 was confirmed as (+)-pinoresinol-4′-*O*-*β*-D-glucopyranoside by matching it with reference standards. Given the same characteristic fragment ions at 357, 342, 151, and 136 in their MS/MS spectra, these compounds may be classified as furofuran-type lignans and characterized as isomers of (+)-pinoresinol-4′-*O*-*β*-D-glucopyranoside. In addition, Compounds **126** and **163** were recognized as phillyrin and phillygenin in reference to the corresponding standards. Arctiin was selected as a representative compound to illustrate the fragmentation patterns of 2,3-dibenzylbutyrolactone type lignans (Figure 3C). The [M−H]^−^ ion at *m*/*z* 579.2081 underwent fragmentation in negative ion mode, yielding product ions at *m*/*z* 371.1529 [M−H−C_6_H_10_O_5_]^−^ and 356.1233 [M−H−C_6_H_10_O_5_−CH_3_]^−^ through the loss of a glucose and a methyl group.

#### 2.1.3. Identification of Cyclohexyl Ethanol Derivatives

Cyclohexane ethanol derivatives (CEDs), a major chemical structure type in FF with a glucose skeleton, are linked to various C6-C2 derivatives at C-1 or C-6 through glycosidic or ester bonds. These compounds exhibit unstable chemical properties, which hinder their isolation and purification [30]. By comparing MS/MS fragment ions with the in-house database, 19 cyclohexane ethanol derivatives were characterized in negative ion mode. The typical cyclohexanol derivative, forsythenside B, in FF produced ions at *m*/*z* 315.1085 [M−H−C_8_H_8_O_4_]^−^, 153.0557 [C_8_H_10_O_3_−H]^−^, 135.0451 [C_8_H_10_O_3_−H−H_2_O]^−^, 167.0350 [C_8_H_8_O_4_−H]^−^, 149.0224 [C_8_H_8_O_4_−H−H_2_O]^−^, and 121.0295 [C_8_H_8_O_4_−H−H_2_O−CO]^−^, resulting from the loss of sugar moieties, H_2_O, and CO (Figure S2B). In comparison with the standards, compounds **8** and **32** were confirmed as cornoside and forsythenside B. Additionally, compounds **4**, **11**, **38**, **45**, **97**, **100**, **103**, and **120** were identified, as shown in Table S3, by searching the molecular formulas of reported compounds in the literature using Compound Discoverer 3.3 (CD) software and matching their fragmentation behaviors.

#### 2.1.4. Identification of Terpenoid

Due to the higher number of fragment ions and stronger responses observed in the positive ion mode for terpenoid compounds (e.g., ursolic acid), their analysis was carried out in this mode. In this study, 71 terpenoids in FF were identified or tentatively characterized. Ursolic acid exhibited a precursor ion at *m*/*z* 457.3576 [M+H]^+^, along with product ions at *m*/*z* 439.3571 [M+H−H_2_O]^+^; and 393.3414 [M+H−2H_2_O−CO]^+^, arising from the sequential loss of water, CO_2_, and CO, respectively (Figure S3A). The ions at *m*/*z* 205.1951 C_15_H_25_^+^ and 189.1638 C_14_H_21_^+^ were also formed through subsequent fragmentation or rearrangement reactions. Through comparison with the standards, compounds **214**, **217**, and **218**, which have the precursor ion [M+H]^+^ at *m*/*z* 457.3676, were identified as betulinic acid, oleanolic acid, and ursolic acid. Moreover, compound **205** was unambiguously determined to be corosolic acid. The abundance of product ions and structural complexity of terpenoids posed challenges in the accurate assignment of ions and structure identification. CD software was used for molecular network analysis, and 30 triterpenoid compounds were identified in ESI^+^ mode (Figure S3B). Each node represents a compound, and the pie chart for each node shows the proportion of the precursor ion peak area.

#### 2.1.5. Identification of Other Compounds

Other components in FF, such as flavonoids, organic acids, and alkaloids, exhibit significant pharmacological effects such as anti-inflammatory, antimicrobial, antioxidant, and anticancer activities [31,32]. Considering the ionization behavior and chemical nature of flavonoids and organic acids, negative ion mode was utilized to improve both the accuracy and sensitivity of the identification process.

Based on the in-house database, nine flavonoids were tentatively identified. Compound **79** was confirmed as rutin. It showed a [M−H]^−^ ion at *m*/*z* 609.1457 (C_27_H_30_O_16_), with fragment ions at *m*/*z* 301.0372 [M−H−C_6_H_10_O_5_−C_6_H_10_O_4_]^−^ and 271.0275 [M−H−C_6_H_10_O_5_−C_6_H_10_O_4_−CH_2_O]^−^, which result from the loss of glucose, rhamnose, and CH_2_O (Figure 3D). An ion related to Retro-Diels-Alder (RDA) cleavage, with *m*/*z* 151.0036 [^1,3^A], was also detected. Based on the fragmentation pattern, compounds **102** and **130** were tentatively assigned as kaempferol 3-*O*-rutinoside and quercetin.

Additionally, hydroxytyrosol (**15**) and caffeic acid (**33**) are typical representatives of organic acids involved in the biosynthesis of phenylethanol glycosides in FF [2]. These organic acids are crucial intermediates in the metabolic pathway, contributing to the formation of phenylethanol glycosides, which possess various bioactivities.

### 2.2. Multivariate Statistical Analysis of Non-Volatile Compounds

**Figure 4:**
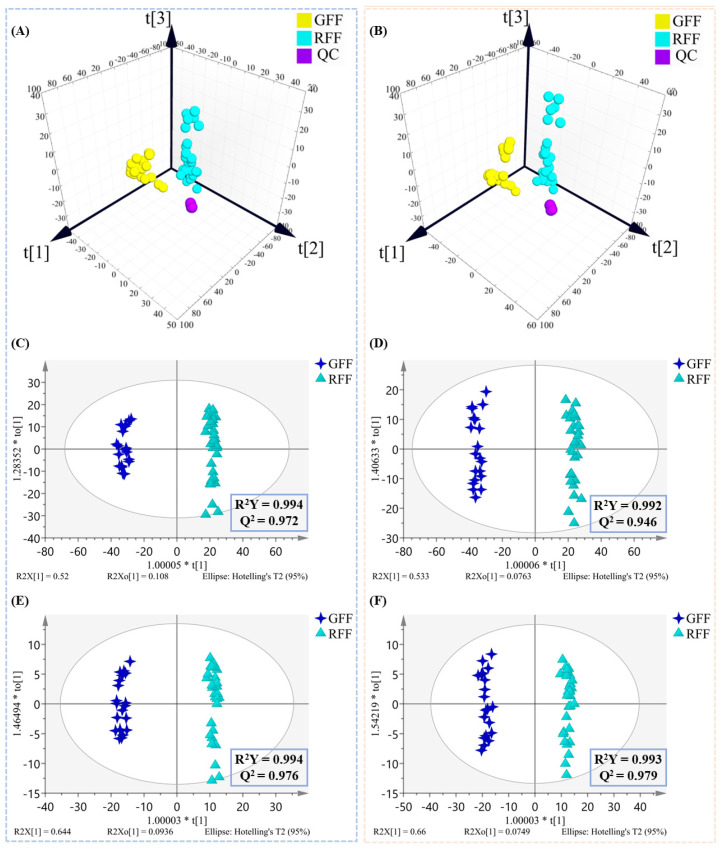
3D PCA score plots of GFF and RFF samples: (**A**) ESI^+^ mode with 8524 features and (**B**) ESI^−^ mode with 12,496 features; OPLS-DA score plots of GFF and RFF samples: (**C**) ESI^+^ mode with 8524 features and (**D**) ESI^−^ mode with 12,496 features; OPLS-DA score plots of GFF and RFF samples: (**E**) ESI^+^ mode with 798 features and (**F**) ESI^−^ mode with 1071 features.

Given that positive and negative ionization modes may result in different metabolite profiles, analyses for ESI^+^ and ESI^−^ data were performed separately to ensure proper handling of the data from each mode. This approach enabled the identification of the specific contributions of each ionization mode to the metabolic differences between GFF and RFF samples, providing a more comprehensive and accurate view of the metabolite variations. CD software was used to process the raw LC-MS data, yielding 8524 and 12,496 mass spectrometric features in positive (ESI^+^) and negative (ESI^−^) ion modes, respectively. The generated data matrix, which contained *m*/*z* values, retention times, and normalized peak areas, was then imported into SIMCA 14.1 for chemometric analysis. As shown in the 3D PCA score plot (Figure 4A,B), the QC samples exhibited tight clustering in both ESI^+^ and ESI^−^ modes, indicating excellent instrument stability and reproducibility. For ESI^+^ (Figure 4A), the first three principal components (PCs) explained 53.8%, 14.9%, and 7.5% of the total variance, respectively, yielding a cumulative interpretation rate (R^2^Xcum) of 83.3%. In ESI^−^ (Figure 4B), the corresponding values were 51.7%, 16.9%, and 9.8%, with a total variance explanation of 89.8%. The explanation rate of ESI^−^ (89.8%) was higher than that of ESI^+^ (83.3%), which reflects greater relative concentration changes between GFF and RFF in metabolites. The OPLS-DA model showed a clear separation between GFF and RFF in both ionization modes (Figure 4C,D), indicating significant differences in their chemical compositions. Furthermore, VIP (Variable Importance in Projection) values were applied to determine metabolites with significant contributions to sample discrimination.

Due to the high-dimensional nature of the LC-MS data, the VIP values could not effectively screen differential metabolites. Therefore, the Result Filter function in CD software was used to remove irrelevant noise, enhancing the identification accuracy of differential compounds. A total of 798 features in ESI^+^ and 1071 features in ESI^−^ were chosen for further OPLS-DA analysis based on the following filtering criteria: Peak rating ≥ 6, RSD QC area ≤ 30%, adjusted *p*-value < 0.01 for any ratio, and a normalized area value for each file. The OPLS-DA models, as shown in Figure 4E,F, demonstrated that the GFF and RFF groups could still be significantly distinguished in ESI^+^ and ESI^−^, and the model parameters (R^2^Y and Q^2^) exhibited good explanatory and predictive ability. Additionally, the 200 permutation tests (Figure S4A,B) indicated that no overfitting phenomenon was observed. These results indicate that the filtering criteria effectively excluded features with low peak quality or unreliable values, without affecting the classification results.

A total of 217 and 353 characteristic peaks were screened in ESI^+^ and ESI^−^, respectively, based on VIP > 1 and *p*-value < 0.05, facilitating the differentiation between GFF and RFF samples. Features without MS/MS spectra were excluded, and differential metabolites were identified based on retention time, *m*/*z*, and fragment ions, as detailed in Figure 1. Finally, 43 differential compounds were identified, with detailed information presented in Table 1. The main differential compounds included 13 phenylethyl glycosides, 8 lignans, 8 iridoids, 6 cyclohexyl alcohol derivatives, 3 terpenes, 3 organic acids, 1 flavonoid, and 1 alkaloid. The clustering heatmap, based on chromatographic peak areas, displays the distribution of 43 differential metabolites in GFF and RFF (Figure S5). The results indicated that most compounds, such as cornoside, isoforsythiaside, and forsythoside A, are more abundant in GFF than in RFF, which is consistent with the findings of Jia et al. [2]. Compared to NMR analysis, the differential compounds identified by LC-MS did not include phillyrin and rutin. This may be related to the ionization efficiency of these compounds and their relatively small differences in GFF and RFF samples. Although the differences observed in the heatmap reflect relative variations in metabolite levels between the sample groups rather than absolute concentration differences, they still offer important insights into the differences between GFF and RFF.

Additionally, the relative concentrations of S-suspensaside methyl ether, forsyshiyanine B, esculentic acid, 18-*β*-glycyrrhetinic acid, and maslinic acid were higher in RFF compared to GFF. This finding could be associated with the anti-inflammatory and mass-dispersing properties of RFF in clinical applications. Forsythianine B, an alkaloid with a rare skeleton structure, exhibits antiviral activities against influenza A virus and respiratory syncytial virus, as well as anti-inflammatory properties [33]. Previous studies have shown that the terpenoid compounds esculentic acid, 18-*β*-glycyrrhetinic acid, and maslinic acid possess significant anti-inflammatory, antitumor, and antioxidant properties [34,35,36]. Wang et al. demonstrated that 18-*β*-glycyrrhetinic acid exerts strong antitumor activity against colorectal cancer by suppressing cell proliferation and migration [35]. Earlier research has mainly explored the variations in phenylethanoid glycosides, lignans, and flavonoids between GFF and RFF [37]. Our findings reveal that terpenoids also play a crucial role in their pharmacological disparities, underscoring their impact on chemical diversity and bioactivity.

### 2.3. Characterization of the Volatile Metabolites in GFF and RFF

**Figure 5:**
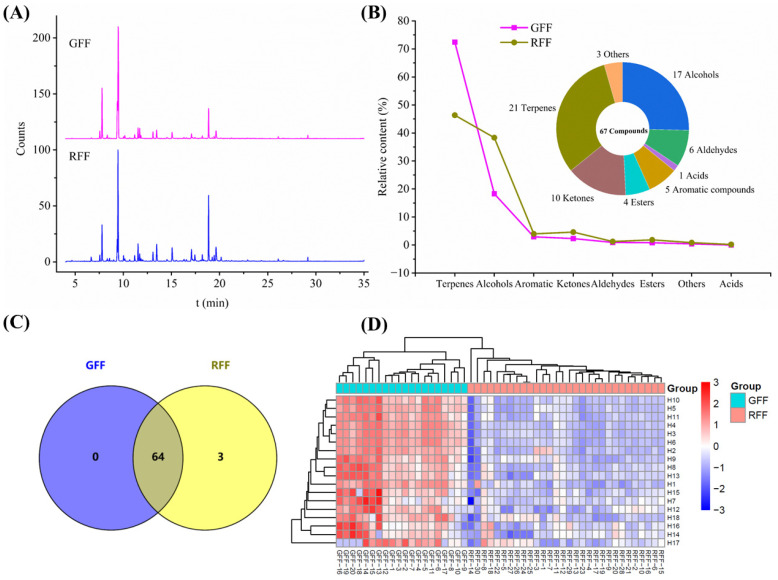
(**A**) Total Ion Chromatograms (TICs) from HS-GC-MS analysis of GFF and RFF samples; (**B**) type composition of volatile compounds and Venn plot (**C**) in GFF and RFF; (**D**) heatmap visualization of 18 differential volatile compounds.

The Total Ion Chromatograms (TICs) obtained by HS-GC-MS analysis clearly reveal significant differences between the GFF and RFF samples (Figure 5A). Sixty-seven volatile compounds were identified based on reference standards, the NIST 17.0 database, and the literature [38,39,40], comprising 21 terpenes, 17 alcohols, 10 ketones, 6 aldehydes, 5 aromatics, 4 esters, 1 acid, and 3 others (Figure 5B). The Venn plot (Figure 5C) showed that 64 volatile components were common to both GFF and RFF. Hexanoic acid, 1-hexanol, and 4-methylenecyclohexanone were only detected in RFF.

The differences in volatile compounds between GFF and RFF may be influenced by multiple factors, including variety, maturity stage, harvest time, drying and storage conditions, and enzymatic and non-enzymatic reactions [41,42,43]. Specifically, it was found that GFF, harvested at an earlier stage, exhibits a higher relative abundance of terpenes (Figure 5B). In contrast, RFF, harvested at full maturity, shows a reduction in terpenes and an increase in alcohols and ketones. These changes are closely related to metabolic transitions during fruit maturation, the regulation of metabolic pathways, and processing methods [44,45]. *β*-Pinene, *α*-pinene, sabinene, and terpinen-4-ol are the major volatile components, with relative contents exceeding 5% in both GFF and RFF samples (Table 2). Moreover, *β*-pinene was the most abundant compound in both GFF and RFF, which is consistent with previous studies [38]. However, the relative content of *β*-pinene significantly decreased during maturation, from 39.96% ± 1.65% in GFF to 23.52% ± 2.64% in RFF. This change may be due to alterations in the terpenoid metabolic pathway of RFF fruits during maturation, such as reduced monoterpene synthase (TPS) activity or increased activity of downstream oxidases, leading to the degradation or transformation of terpene compounds [46].

Furthermore, the proportion of terpenes in GFF is 72.34%, significantly higher than in RFF (46.30%). This trend suggests that terpene synthesis is more active during early fruit development. As the fruit matures, the secondary metabolic pathways gradually shift toward other metabolic products, such as alcohols and ketones, which may play an important role in fruit maturation and adaptation to environmental changes [47]. Recent research indicated that the accumulation of certain alcohols and ketones during fruit maturation could be associated with cell wall degradation, flavor formation, and stress regulation [48]. Therefore, the differences in volatile components between GFF and RFF could be attributed to metabolic changes, enzymatic transformations, and growth conditions during fruit maturation.

### 2.4. Multivariate Statistical Analysis of Volatile Compounds

**Figure 6:**
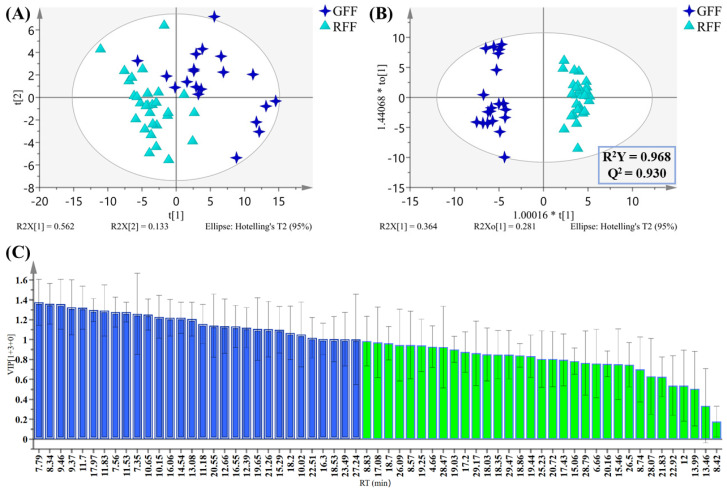
Score plots of the PCA model (**A**) and OPLS-DA model (**B**); VIP plots (**C**) based on 67 compounds identified by HS-GC-MS.

HS-GC-MS qualitative analysis indicated that the types of volatile compounds in GFF and RFF samples are similar, but their relative concentrations vary significantly. To better visualize these differences and identify the differential compounds, PCA and OPLS-DA analyses were performed using the 67 identified volatile compounds. From the PCA score plot (Figure 6A), it can be seen that the first two PCs explain about 69.5% of the total variance, but the GFF and RFF samples did not fully separate. However, the OPLS-DA score plot (Figure 6B) exhibited a clear separation between GFF and RFF samples, consistent with the PCA models. Model parameters (R^2^X = 0.785, R^2^Y = 0.968, Q^2^ = 0.93) indicated robust explanatory and predictive abilities. Furthermore, 200 permutation tests confirmed the absence of overfitting. The VIP values of 67 compounds are displayed in Figure 6C, and 18 volatile compounds (VIP > 1 and *p*-value < 0.05) were chosen as potential markers to distinguish GFF from RFF (Table S4). The cluster heatmap analysis (Figure 4D) reveals a clear separation between GFF and RFF samples, indicating distinct differences in the volatile components between GFF and RFF. The analysis of these characteristic metabolites revealed that the accumulation of ketones, alcohols, aldehydes, and esters was higher in RFF compared to GFF, with prominent compounds such as sabinone, o-cymene, and terpinolene. In contrast, the levels of terpene compounds, including *α*-thujene, *α*-pinene, (R)-isocarvestrene, camphene, cyclene, sabinene, *β*-pinene, and eucalyptol, were significantly reduced.

### 2.5. Discriminant Ability Assessment of Differential Compounds

**Figure 7:**
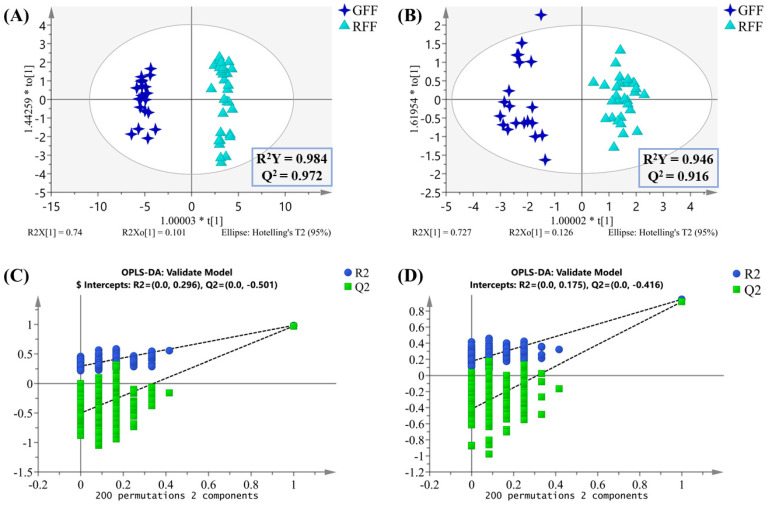
OPLS-DA score plots based on the screened 43 differential metabolites identified by LC-MS (**A**) and 18 differential metabolites identified by HS-GC-MS (**B**); 200 times permutation tests of the OPLS-DA model for differential metabolites identified by LC-MS (**C**) and HS-GC-MS (**D**).

To assess the discriminative power of the selected differential compounds, both supervised OPLS-DA and unsupervised HCA methods were applied. The two new OPLS-DA models in Figure 7A,B demonstrate comparable effectiveness to the original models (Figure 4C,D) in distinguishing the samples. Additionally, the OPLS-DA model results show that LC-MS outperforms HS-GC-MS in sample distribution, suggesting that LC-MS provides a higher resolution and greater application potential, particularly offering unique advantages in the analysis of complex samples. Moreover, these new models exhibit strong statistical parameters, confirming their reliable fitting and predictive performance. The permutation test confirms the robustness and strong predictive capabilities of the two OPLS-DA models (Figure 4C,D). Distinct clustering results can be observed by HCA (Hierarchical Clustering Analysis, Figure S6B), which are consistent with the OPLS-DA results. Overall, the differential compounds screened by the untargeted metabolomics approach using LC-MS and HS-GC-MS provide strong support for distinguishing between GFF and RFF.

### 2.6. Mid-Level Data Fusion of LC-MS and HS-GC-MS 

LC-MS is capable of providing information regarding both polar and non-polar compounds. In contrast, GC-MS primarily focuses on volatile compounds. Nevertheless, variations in sensitivity, detection limits, and sample preparation among different instruments can lead to disparate results. By integrating data fusion methods with multiple analytical techniques, a more extensive metabolite profile can be obtained [21,49]. Rivera-Pérez et al. demonstrated that mid-level data fusion, combining UHPLC-HRMS, GC-HRMS, and ^1^H NMR, enhanced the classification accuracy of black pepper from 92% to 100%, highlighting its potential for metabolomics-based authentication and quality control [49].

**Figure 8:**
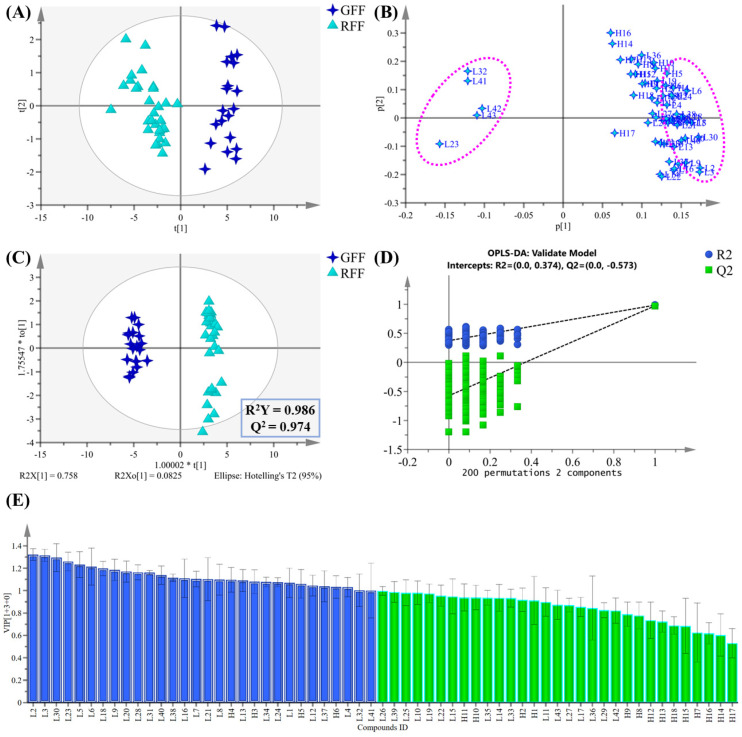
Score plots (**A**) and loading plots (**B**) of the PCA model; score plots of the OPLS-DA model (**C**); 200 times permutation tests (**D**); VIP plots (**E**) based on the mid-level fusion method.

In this study, a mid-level data fusion method was used to efficiently combine the results from LC-MS and HS-GC-MS analyses. This strategy facilitated a comprehensive understanding of the volatile and non-volatile constituents in the FF samples. The fused dataset consisted of 43 non-volatile differential metabolites identified by LC-MS and 18 volatile differential metabolites identified by HS-GC-MS. After min-max normalization, the data matrix was uploaded to SIMCA 14.1 software, followed by log transformation and Pareto scaling before PCA and OPLS-DA analysis. The PCA score plot (Figure 8A) reveals that GFF and RFF are clearly separated along the first two principal components (PC1 and PC2), suggesting significant differences in metabolite composition between the groups. The loading plot (Figure 8B) shows the contribution of key metabolites to the sample separation. Metabolites farther from the origin have a greater impact on the group differences. For example, compounds L2 (hydroxytyrosol 1-*O*-glucoside), L3 (cornoside), L23 (S-suspensaside methyl ether), L30 (forsythenside L), L32 (forsyshiyanine B), and L41 (esculentic acid) contribute significantly to the first two principal components, suggesting their important role in the differences between groups. The findings align with the OPLS-DA model, where the VIP values of these five compounds exceed 1.0 (Figure 8E). The OPLS-DA model with mid-level data fusion showed improved separation between GFF and RFF samples (Figure 8C). Moreover, the model exhibited superior performance, with reliable goodness-of-fit (R^2^Y = 0.986) and predictability (Q^2^ = 0.974), outperforming the single HS-GC-MS technique (R^2^Y = 0.968, Q^2^ = 0.930) (Table S5).

Additionally, mid-level data fusion was applied to further screen markers in order to identify metabolites with higher specificity and representativeness. Using VIP > 1 and *p*-value < 0.05 as criteria, 30 significantly different compounds were identified between GFF and RFF (Figure 8E). Among them, 26 non-volatile compounds were identified through LC-MS, with the top 10 compounds based on VIP values, including hydroxytyrosol-1-*O*-glucoside, cornoside, forsythenside L, S-suspensaside methyl ether, vanilloloside, forsythoside D, forsythenside B, (+)-8-hydroxypinoresinol 4-*O*-*β*-D-glucopyranoside, salidroside, and forsythialanside E. Additionally, four volatile compounds, including *α*-pinene, camphene, sabinene, and *β*-pinene, were selected through HS-GC-MS analysis. By reducing the 61 differential compounds initially identified through single techniques to 30 compounds selected through mid-level data fusion, this process effectively eliminated irrelevant variables and maintained excellent predictive performance.

Furthermore, the differential compounds identified through mid-level data fusion mainly originate from non-volatile compounds from LC-MS analysis. This may be attributed to the lower content of volatile compounds in GFF and RFF, compared to non-volatile components. This phenomenon might be a consequence of the volatile nature of these compounds, which makes them prone to loss during long-term storage or extraction processes, leading to lower concentrations [43]. Despite the relatively high concentrations of volatile compounds in GFF and RFF, HS-GC-MS still provides valuable insights, particularly in revealing the sensory characteristics and potential biological activity of the medicinal materials. For example, volatile compounds such as *α*-pinene, camphene, and sabinene, which were detected, are closely related to the flavor, aroma, and potential biological activity (e.g., anti-inflammatory and antimicrobial) of the medicinal material [50,51].

## 3. Materials and Methods

### 3.1. Chemicals and Materials

The ultrapure water was sourced from Guangzhou Watsons Food and Beverage Co., Ltd. in Guangzhou, China. Methanol and acetonitrile (LC-MS grade), as well as the n-alkane standards (C_8_-C_25_), were purchased from Merck in Darmstadt, Germany. Formic acid (LC-grade) was supplied by Anpel Laboratory Technologies Inc. (Shanghai, China). Other chemicals and solvents were sourced from Sinopharm (Shanghai, China). Twenty-one reference standards from Shanghai Shidande Standard Technical Service Co., Ltd. were used for LC-MS analysis. Eight reference standards from Shanghai Aladdin Biochemical Technology Co., Ltd. (Shanghai, China) were used for HS-GC-MS analysis (Table S2). All reference standards met analytical purity requirements.

Fifty batches of FF were collected from commercial suppliers, with detailed information provided in Table S3. The samples of FF were stored at the School of Pharmacy, Hunan University of Chinese Medicine, China.

### 3.2. UPLC-Q/Orbitrap MS Analysis

#### 3.2.1. Preparation of the Sample Solution

A 50 mg sample powder was precisely weighed and extracted ultrasonically with 50 mL of 70% methanol for 30 min. After centrifugation at 13,000 rpm for 10 min, the supernatant was separated and used for LC-MS analysis. Equal volumes of supernatant from different sample batches were combined to prepare the quality control (QC) samples. Standards were dissolved in 70% methanol to prepare stock solutions, which were then mixed in batches to identify isomers. All extracts were kept at −40 °C until analysis.

#### 3.2.2. Data Acquisition

The chromatographic separation was conducted on a Hypersil GOLD™ Aq-C18 column (100 × 2.1 mm, 1.9 μm) provided by Thermo Fisher Scientific (Waltham, MA, USA). The mobile phase consisted of 0.1% formic acid in water (A) and acetonitrile (B), with a flow rate of 0.3 mL/min, and the gradient was set as follows: 0–5 min, 3–5% A; 5–10 min, 5–15% A; 10–15 min, 15–17% A; 15–20 min, 17% A; 20–35 min, 17–50% A; 35–45 min, 50–65% A; 45–55 min, 65–95% A; 55–65 min, 95–98% A. Before injecting 2 μL of the sample solution for analysis, the column was maintained at 30 °C and equilibrated for 5 min.

Mass spectrometry was executed on an Orbitrap Exploris 120 system (Thermo Fisher Scientific, Waltham, MA, USA), utilizing heated electrospray ionization (H-ESI) in both positive (+3500 V) and negative (−3000 V) ion modes. The ion transfer tube had a temperature of 325 °C, and the vaporizer was at 350 °C. The scan range was defined from 100 to 1200 Da, with an intensity threshold set at 2 × 10^5^. Fragmentation was performed in ddMS^2^ mode at 15,000 resolutions with stepped HCD collision energies of 30, 60, and 90 eV. To verify the stability of the instrument, QC samples were examined initially and after every eighth injection.

#### 3.2.3. Data Pre-Processing

The LC-MS data were analyzed in Compound Discoverer 3.3 (CD) with an untargeted metabolomics workflow. The workflow included peak extraction, baseline correction, retention time alignment, background subtraction, and peak area normalization. Compounds were identified within 1–60 min retention time, with a 5 ppm mass tolerance and a minimum intensity of 100,000. The analysis was conducted separately for positive and negative ion modes. Median normalization of peak areas was applied after background filtering and gap filling. The analysis was based on relative concentrations, as the peak area normalization method reflects the relative abundance of metabolites. Absolute concentrations of metabolites were not measured or considered in this study.

An in-house FF database was developed from the literature (e.g., Scifinder, PubMed, CNKI) and imported into CD software. The identification and characterization strategy for FF chemical constituents is shown in Figure 1. Elemental composition was predicted based on exact masses. Mass Frontier 8.0 was used to analyze the fragmentation patterns of reference compounds. The diagnostic product ions (DPIs) and neutral losses (NLs) of different types of compounds, based on the fragmentation patterns of reference compounds, were summarized. The differentiation of isomers was achieved by calculating ClogP values with ChemBioDraw Ultra 14.0.

### 3.3. HS-GC-MS Analysis

#### 3.3.1. Instruments and Conditions

The sample powder (50 mg) was accurately weighed and placed in a 20 mL headspace vial, sealed, and then inserted into the 7697A Headspace Sampler (Agilent, Santa Clara, CA, USA). An Agilent 7890/7000A GC-QQQ/MS system (Agilent, Santa Clara, CA, USA) was used for compound identification. The vial was heated to 120 °C and equilibrated for 30 min. The quantitative loop was maintained at 130 °C, while the transfer line was set to 140 °C. The balance time for the quantitative loop was 0.05 min. A 1000 μL injection volume was used, with 0.5 min for sample retrieval, and a 40-min GC cycle time.

An Agilent HP-5MS Capillary GC column (30 m × 0.25 mm × 0.25 µm) was used. The sample injection was carried out at 250 °C in 1:20 split modes, with a solvent delay of 3 min. The temperature program began at 50 °C for 3 min, then ramped up to 80 °C at 3 °C/min for 2 min. After that, it increased to 120 °C at 5 °C/min, held for 2 min, and eventually rose to 240 °C at 12 °C/min. After the run, the temperature was held at 280 °C for 3 min, with a total run time of 38 min. The electron ionization source was kept at 230 °C with an ionization voltage of 70 eV. Full-scan mode (*m*/*z* 50–550) was used for data acquisition, with the ion source and quadrupole maintained at 250 °C and 150 °C, respectively. n-Alkane standards (C_8_-C_25_) were injected for analysis.

#### 3.3.2. Data Processing

The raw HS-GC-MS data were analyzed with the Agilent MassHunter Unknowns program (Version 10.1), and compounds were identified by matching with the NIST 17.0 library (scores > 80%). Retention indices (RIs) were calculated using n-alkanes (C_8_-C_25_) and compared to the NIST database values for the same column, with identifications accepted when RI differences were <10. Reference compounds were employed to confirm the identifications. The relative content of compounds was calculated using the peak area normalization method and expressed as the mean ± SD (standard deviation).

### 3.4. Chemometric Analysis

For LC-MS analysis, chemometric analysis was performed on raw features rather than identified compounds due to the complexity of the data. LC-MS generates a large number of ions, and direct analysis of identified compounds alone may not adequately capture the full metabolite profile. HS-GC-MS provides a direct and reliable analytical approach, simplifying data processing and ensuring accurate compound identification and quantification. Therefore, identified compounds are used for chemometric analysis in HS-GC-MS analysis. The peak areas of the extracted features or compounds were imported into SIMCA 14.1 software (Umetrics, Malmo, Sweden) for chemometric analysis. Data were Log-transformed and Pareto-scaled before performing PCA and OPLS-DA analysis. R^2^Y and Q^2^ were used as validation parameters for the OPLS-DA model, with 200 permutation tests to avoid overfitting. Differential compounds were selected with VIP ≥ 1 and *p*-value < 0.05. In addition, fold change (FC) analysis was applied to compare compound abundances between groups. Cluster heatmaps were visualized using the Bioinformatics platform, and hierarchical clustering of metabolite biomarkers was performed with MetaboAnalyst 5.0.

### 3.5. Mid-Level Data Fusion Strategies

Mid-level data fusion was applied to integrate LC-MS and HS-GC-MS datasets for differentiating the ripening stages of FF, aiming to assess metabolite information and improve prediction accuracy. VIP scores from OPLS-DA models and *t*-test *p*-values obtained from multiple platforms were used to identify discriminative compounds, which were subsequently integrated into a new matrix for classification. Min-max normalization was applied to address scale differences, followed by auto-scaling of the mid-level matrix before modeling.

## 4. Conclusions

In this study, a systematic analysis of GFF and RFF compounds was performed using LC-MS and HS-GC-MS, identifying 237 non-volatile compounds and 67 volatile compounds. High-resolution LC-MS offers superior sensitivity and broader metabolite coverage, allowing for the simultaneous detection of both polar and non-polar compounds while providing structural information, making it particularly advantageous for the identification of non-volatile metabolites. HS-GC-MS features low sample consumption and rapid separation and detection of volatile compounds without additional extraction. However, LC-MS has limitations in detecting volatile compounds, and HS-GC-MS has lower sensitivity for non-volatile compounds. Therefore, integrating the complementary strengths of both techniques is crucial for improving the accuracy and comprehensiveness of the analysis.

A mid-level data fusion strategy was employed to overcome the potential information gaps or limitations of single techniques, thereby enhancing the reliability and representativeness of the analysis. Using VIP > 1 and *p*-value < 0.05 as criteria, 30 differential metabolites, including 26 non-volatile and 4 volatile compounds, were identified. Among them, only S-suspensaside methyl ether, forsyshiyanine B, and esculentic acid showed higher relative concentrations in RFF than in GFF. Although a higher relative concentration does not necessarily indicate a higher absolute concentration, the relative concentrations of metabolites can still provide valuable insights into the differences between GFF and RFF.

This study provides scientific evidence for the quality assessment, authenticity identification, and pharmacological research of GFF and RFF, demonstrating the potential of data fusion in Traditional Chinese Medicine. However, relying solely on chemical composition analysis is insufficient to fully explain the pharmacological differences, and future studies could combine in vitro or vivo bioactivity assays and network pharmacology methods to further explore the mechanisms of action.

## Figures and Tables

**Table 1:** Detailed information on the 43 differential compounds identified by LC-MS.

No.	Compound Name	VIP Score	RT (min)	Adduct	Formula	*m*/*z*	Error (ppm)	MS/MS
L1	Quinic acid	1.24	0.98	[M−H]^−^	C_7_H_12_O_6_	191.0560	−0.74	173.0463, 127.0405
L2	Hydroxytyrosol 1-*O*-glucoside	1.52	1.56	[M−H]^−^	C_14_H_20_O_8_	315.1085	−0.41	153.0559, 135.0457, 89.0246
L3	Cornoside	1.52	2.52	[M−H]^−^	C_14_H_20_O_8_	315.1084	−1.85	153.0571, 135.0455, 119.0350, 113.0244
L4	Rengynic acid-1-*O*-*β*-D-glucopyranoside	1.24	3.46	[M−H]^−^	C_14_H_24_O_9_	335.1346	−0.34	179.0579, 161.0463, 131.0347, 119.0352
L5	Vanilloloside	1.4	3.92	[M−H]^−^	C_14_H_20_O_8_	315.1085	−0.28	153.0554, 135.0459, 119.0353, 101.0250
L6	Forsythoside D	1.43	5.49	[M−H]^−^	C_20_H_30_O_13_	477.1605	−1.71	449.4005, 315.1103, 179.0560, 161.0463, 135.0455
L7	Adoxosidic acid	1.28	6.01	[M−H]^−^	C_16_H_24_O_10_	375.1284	−3.20	337.1743, 213.0771, 169.0872, 151.0768, 125.0611
L8	Forsythide	1.23	6.28	[M−H]^−^	C_16_H_22_O_11_	389.1077	−3.05	345.1214, 183.0665, 165.0560, 121.0662, 119.0355
L9	Salidroside	1.35	6.66	[M−H]^−^	C_14_H_20_O_7_	299.1135	−2.30	119.0350, 101.0248
L10	Rebouoside B	1.11	6.76	[M−H]^−^	C_19_H_28_O_12_	447.1499	−2.07	315.1107, 191.0566, 149.0456, 135.0450, 131.0353
L11	Forsythoside E	1.02	7.19	[M−H]^−^	C_20_H_30_O_12_	461.1655	−2.05	315.1081, 163.0613, 153.0561, 135.0454, 131.0353
L12	Darendoside A	1.26	7.76	[M−H]^−^	C_19_H_28_O_11_	431.1550	−1.99	299.1140, 191.0568, 149.0460, 137.0603, 131.0353
L13	Forsythenside B	1.24	7.86	[M−H]^−^	C_22_H_26_O_11_	465.1396	−1.15	161.0456, 153.0552, 149.0247, 135.0454
L14	4-*O*-*p*-Coumaroylquinic acid	1.1	9.33	[M−H]^−^	C_16_H_18_O_8_	337.0929	−0.05	191.0565, 173.0457, 163.0402, 145.0300
L15	Rengyoside D	1.13	9.39	[M−H]^−^	C_22_H_30_O_11_	469.1709	−1.42	193.0505, 161.0617, 151.0403, 133.0296, 123.0459
L16	Hastatoside	1.19	10.05	[M−H]^−^	C_17_H_24_O_11_	403.1236	−2.53	371.0999, 327.1099, 191.0359, 179.0715, 137.0610
L17	Calceolarioside A	1.02	10.89	[M−H]^−^	C_23_H_26_O_11_	477.1395	−1.61	179.0354, 161.0250, 135.0456, 133.0298
L18	(+)-8-Hydroxypinoresinol 4-*O*-*β*-D-glucopyranoside	1.2	11.27	[M−H]^−^	C_26_H_32_O_11_	535.1816	−0.66	373.1295, 343.1180, 313.1063, 179.0718, 163.0406, 151.0397, 109.0295
L19	Forsythiayanoside B	1.44	11.47	[M−H]^−^	C_27_H_36_O_12_	551.2130	−0.64	359.1494, 191.0721, 163.0404
L20	Forsythialanside E	1.19	11.69	[M−H]^−^	C_26_H_32_O_11_	581.1874	−0.31	373.1261, 343.1176, 163.0407
L21	Calceolarioside C	1.28	11.97	[M−H]^−^	C_28_H_34_O_15_	609.1819	−1.01	447.1505, 315.1081, 179.0351, 161.0245, 153.0568
L22	Isoforsythiaside	1.1	12.36	[M−H]^−^	C_29_H_36_O_15_	623.1979	−0.39	461.1692, 443.1567, 179.0353, 161.0246, 135.0454
L23	S-Suspensaside methyl ether	1.54	12.67	[M−H]^−^	C_30_H_38_O_16_	653.2085	−0.33	621.1834, 179.0354 161.0246, 151.0404, 135.0454
L24	Plantainoside A	1.3	12.93	[M−H]^−^	C_23_H_26_O_11_	477.1395	−1.44	315.1104, 179.0346, 161.0247, 135.0455, 133.0298
L25	Adoxosidic acid-6′-oleuroperic ester	1.17	13.41	[M−H]^−^	C_30_H_36_O_16_	651.1928	−0.39	427.1253, 179.0351, 163.0611, 161.0246, 135.0453
L26	Forsythoside A	1.19	13.54	[M−H]^−^	C_29_H_36_O_15_	623.1978	−0.50	461.1658, 315.1178, 179.0352, 161.0245, 135.0453
L27	Acteoside	1.05	14.15	[M−H]^−^	C_29_H_36_O_15_	623.1978	−0.50	461.1663, 315.1077, 179.0348, 161.0245, 153.0556
L28	Calceolarioside B	1.35	14.21	[M−H]^−^	C_23_H_26_O_11_	477.1395	−1.47	315.1085, 179.0352, 161.0246, 135.0452, 133.0297
L29	Kaempferol 3-*O*-rutinoside	1.02	14.95	[M−H]^−^	C_27_H_30_O_15_	593.1511	−0.12	447.1546, 285.0403, 163.0035, 151.0037, 135.0085
L30	Forsythenside L	1.57	15.12	[M−H]^−^	C_20_H_28_O_11_	443.1551	−1.86	427.8166, 149.0611
L31	(+)-Pinoresinol 4′-*O*-*β*-D-glucopyranoside	1.39	15.42	[M−H]^−^	C_26_H_32_O_11_	519.1867	−0.98	357.1334, 342.1089, 311.1313, 163.0404, 161.0261, 151.0402, 136.0166
L32	Forsyshiyanine B	1.09	15.98	[M+H]^+^	C_18_H_17_NO_3_	296.1281	−0.10	132.0807, 117.0573
L33	Isoacteoside	1.09	16.51	[M−H]^−^	C_29_H_36_O_15_	623.1978	−0.48	461.1663, 179.0351, 161.0245, 153.0553, 135.0453
L34	Fraxiresinol-4′-*O*-*β*-D-glucopyranoside	1.26	18.73	[M−H]^−^	C_27_H_34_O_13_	565.1925	−0.37	357.1336, 327.1258, 151.0403, 137.0243, 135.0451
L35	Matairesinoside	1.06	20.40	[M−H]^−^	C_26_H_32_O_11_	519.1867	−0.91	357.1335, 342.1098, 137.0614, 122.0375
L36	Forsydoitriside A	1.08	22.50	[M−H]^−^	C_48_H_68_O_28_	1091.3822	−0.24	929.3305, 883.8681, 733.2557, 715.2502, 571.2033, 553.1916, 445.1343, 375.1285, 357.1173, 151.0765
L37	Suspenoidside A	1.19	22.79	[M−H]^−^	C_25_H_30_O_12_	521.1660	−0.82	315.1242, 163.0400, 149.0121, 145.0293, 119.0502
L38	Acanthoside B	1.33	23.76	[M−H]^−^	C_28_H_36_O_13_	579.2081	−0.41	371.1505, 356.1262, 121.0295
L39	Suspenoidside E	1.24	23.99	[M−H]^−^	C_26_H_32_O_13_	551.1767	−0.62	345.1339, 327.1238, 193.0508, 179.0366, 149.0614
L40	Suspenoidsides D	1.35	25.81	[M−H]^−^	C_25_H_30_O_13_	537.1610	−0.68	243.0665, 137.0250, 135.0089, 109.0298, 91.0191
L41	Esculentic acid	1.19	32.25	[M+H]^+^	C_30_H_48_O_5_	489.3574	−0.17	435.3245, 407.3309, 219.1744, 201.1638, 191.1795
L42	18-*β*-Glycyrrhetinic acid	1.04	35.94	[M+H]^+^	C_30_H_46_O_4_	471.3467	−0.33	425.3417, 407.3317, 317.2097, 191.1799, 189.1639
L43	Maslinic acid	1.09	39.15	[M+H]^+^	C_30_H_48_O_4_	473.3626	0.10	437.3422, 427.3572, 357.2803, 315.2331, 189.1631

**Table 2:** The volatile compounds and their relative contents in GFF and RFF samples obtained by HS-GC-MS analysis.

No.	RT ^a^ (min)	Compounds Name	Formula	CAS Number	RI_cal_ ^b^	RI_ref_ ^c^	Class	Relative Content ^d^ (%)	
								(Mean ± SD)	
								GFF	RFF
1	4.66	Furfural	C_5_H_4_O_2_	98-01-1	835	833	Aldehydes	0.25 ± 0.09	0.25 ± 0.11
2	5.68	1-Hexanol	C_6_H_14_O	111-27-3	872	868	Alcohols	-	0.07 ± 0.05
3	6.66	3-Methylcyclopentyl acetate	C_8_H_14_O_2_	24070-70-0	901	905	Esters	0.27 ± 0.05	0.74 ± 0.17
4	7.35	Cyclene	C_10_H_16_	508-32-7	922	925	Terpenes	0.05 ± 0.01	0.04 ± 0.02
5	7.56	*α*-thujene	C_10_H_16_	2867-05-2	929	929	Terpenes	1.92 ± 0.22	1.63 ± 0.74
6	7.79	*α*-Pinene	C_10_H_16_	80-56-8	935	937	Terpenes	13.09 ± 1.12	6.83 ± 1.21
7	8.34	Camphene	C_10_H_16_	79-92-5	950	952	Terpenes	0.92 ± 0.11	0.46 ± 0.07
8	8.42	4-Methylenecyclohexanone	C_7_H_10_O	29648-66-6	952	\	Ketones	-	0.11 ± 0.04
9	8.57	2,4-Thujadiene	C_10_H_14_	36262-09-6	956	956	Others	0.24 ± 0.06	0.45 ± 0.16
10	8.74	(2E)-2-Heptenal	C_7_H_12_O	18829-55-5	960	958	Aldehydes	0.04 ± 0.01	0.09 ± 0.04
11	8.83	Benzaldehyde	C_7_H_6_O	100-52-7	962	962	Aromatic	0.06 ± 0.01	0.12 ± 0.02
12	9.37	Sabinene	C_10_H_16_	3387-41-5	975	974	Terpenes	8.98 ± 1.04	4.88 ± 1.18
13	9.46	*β*-Pinene	C_10_H_16_	127-91-3	977	979	Terpenes	39.96 ± 1.65	23.52 ± 2.64
14	9.84	Hexanoic acid	C_6_H_12_O_2_	142-62-1	985	990	Acids	-	0.2 ± 0.07
15	10.02	6-Methylhept-5-en-2-one	C_8_H_14_O	110-93-0	989	986	Ketones	0.6 ± 0.11	1.07 ± 0.15
16	10.15	*β*-Myrcene	C_10_H_16_	123-35-3	992	991	Terpenes	0.72 ± 0.18	0.69 ± 0.08
17	10.65	*α*-Phellandrene	C_10_H_16_	99-83-2	1003	1005	Terpenes	0.3 ± 0.04	0.4 ± 0.05
18	11.18	*α*-Terpinene	C_10_H_16_	99-86-5	1016	1017	Terpenes	1.15 ± 0.14	1.52 ± 0.37
19	11.53	o-Cymene	C_10_H_14_	527-84-4	1024	1022	Aromatic	2.59 ± 0.31	3.23 ± 0.57
20	11.70	(R)-Isocarvestrene	C_10_H_16_	1461-27-4	1028	1027	Terpenes	2.22 ± 0.32	1.83 ± 0.2
21	11.83	Eucalyptol	C_10_H_18_O	470-82-6	1031	1032	Alcohols	0.88 ± 0.08	0.72 ± 0.09
22	12.00	Benzyl alcohol	C_7_H_8_O	100-51-6	1035	1036	Aromatic	0.17 ± 0.02	0.39 ± 0.1
23	12.39	Phenylacetaldehyde	C_8_H_8_O	122-78-1	1044	1045	Aldehydes	0.07 ± 0.01	0.09 ± 0.03
24	12.66	*trans*-Thujenol	C_10_H_16_O	97631-68-0	1049	1039	Alcohols	0.05 ± 0.01	0.05 ± 0.01
25	13.08	*γ*-Terpinene	C_10_H_16_	99-85-4	1058	1060	Terpenes	1.84 ± 0.24	2.38 ± 0.53
26	13.46	(Z)-sabinene hydrate	C_10_H_18_O	15537-55-0	1066	1070	Alcohols	2.33 ± 0.15	5.53 ± 1.68
27	13.99	Benzyl formate	C_8_H_8_O_2_	104-57-4	1076	1080	Esters	0.04 ± 0.02	0.08 ± 0.02
28	14.54	Terpinolene	C_10_H_16_	586-62-9	1086	1088	Terpenes	0.42 ± 0.04	0.59 ± 0.14
29	15.06	(E)-sabinene hydrate	C_10_H_18_O	17699-16-0	1170	1070	Alcohols	1.89 ± 0.18	5.16 ± 1.7
30	15.29	Linalool	C_10_H_18_O	78-70-6	1100	1099	Alcohols	0.09 ± 0.01	0.16 ± 0.04
31	15.46	3,4-Dimethylstyrene	C_10_H_12_	27831-13-6	1104	1100	Others	0.16 ± 0.03	0.39 ± 0.1
32	15.87	Fenchol	C_10_H_16_O	1632-73-1	1114	1113	Alcohols	0.05 ± 0.04	0.18 ± 0.05
33	15.93	Phenylethyl Alcohol	C_8_H_10_O	60-12-8	1115	1116	Aromatic	0.1 ± 0.03	0.2 ± 0.04
34	16.06	Thujone	C_10_H_16_O	546-80-5	1118	1103	Ketones	0.07 ± 0.01	0.1 ± 0.02
35	16.30	4-Terpinenyl acetate	C_12_H_20_O_2_	4821-04-9	1124	1301	Esters	0.35 ± 0.06	0.68 ± 0.11
36	16.55	*α*-Campholenal	C_10_H_16_O	4501-58-0	1130	1125	Aldehydes	0.31 ± 0.06	0.43 ± 0.15
37	17.08	Laevo-pinocarveol	C_10_H_16_O	547-61-5	1142	1139	Alcohols	1.49 ± 0.36	2.91 ± 0.72
38	17.20	(E)-Para-2-menthen-1-ol	C_10_H_18_O	29803-81-4	1144	1140	Alcohols	0.35 ± 0.07	0.84 ± 0.09
39	17.43	Camphene hydrate	C_10_H_18_O	465-31-6	1151	1148	Alcohols	0.5 ± 0.12	1.48 ± 0.45
40	17.97	Sabinone	C_10_H_14_O	67690-48-6	1160	1163	Ketones	0.08 ± 0.02	0.11 ± 0.03
41	18.03	Sabina ketone	C_9_H_14_O	513-20-2	1163	1156	Ketones	0.1 ± 0.03	0.27 ± 0.07
42	18.20	Pinocarvone	C_10_H_14_O	30460-92-5	1166	1164	Ketones	0.79 ± 0.18	1.25 ± 0.45
43	18.35	endo-Borneol	C_10_H_18_O	507-70-0	1169	1167	Alcohols	0.16 ± 0.03	0.43 ± 0.1
44	18.53	*cis*-Sabinol	C_10_H_16_O	3310-02-9	1172	1175	Alcohols	0.1 ± 0.03	0.16 ± 0.03
45	18.70	Pinocamphone	C_10_H_16_O	15358-88-0	1176	1173	Ketones	0.05 ± 0.03	0.11 ± 0.04
46	18.86	Terpinen-4-ol	C_10_H_18_O	562-74-3	1180	1177	Alcohols	6.77 ± 1.13	15.31 ± 1.67
47	19.03	Myrtanal	C_10_H_16_O	4764-14-1	1183	1188	Aldehydes	0.11 ± 0.02	0.17 ± 0.04
48	19.25	4-Isopropyl-2-cyclohexen-1-one	C_9_H_14_O	500-02-7	1187	1184	Ketones	0.36 ± 0.06	0.8 ± 0.21
49	19.44	*α*-Terpineol	C_10_H_18_O	98-55-5	1191	1189	Alcohols	0.51 ± 0.12	1.19 ± 0.25
50	19.65	(±)-Myrtenol	C_10_H_16_O	515-00-4	1195	1195	Alcohols	2.99 ± 0.76	3.96 ± 1.27
51	20.16	(S)-Verbenone	C_10_H_14_O	80-57-9	1208	1205	Ketones	0.24 ± 0.11	0.85 ± 0.26
52	20.55	Carveol	C_10_H_16_O	99-48-9	1220	1219	Alcohols	0.09 ± 0.02	0.13 ± 0.04
53	20.72	2-Hydroxycineole	C_10_H_18_O_2_	18679-48-6	1224	1228	Alcohols	0.06 ± 0.02	0.14 ± 0.05
54	21.26	Cuminaldehyde	C_10_H_12_O	122-03-2	1241	1239	Aldehydes	0.17 ± 0.04	0.23 ± 0.07
55	21.83	Piperitone	C_10_H_16_O	89-81-6	1257	1253	Ketones	0.03 ± 0.01	0.07 ± 0.03
56	22.51	*β*-Cubebene	C_15_H_24_	13744-15-5	1392	1389	Terpenes	0.06 ± 0.01	0.09 ± 0.03
57	22.92	Bornyl acetate	C_12_H_20_O_2_	5655-61-8	1287	1284	Esters	0.16 ± 0.03	0.34 ± 0.05
58	23.49	3-tert-Butylphenol	C_10_H_14_O	585-34-2	1302	1296	Aromatic	0.03 ± 0.01	0.05 ± 0.02
59	25.23	*α*-Cubebene	C_15_H_24_	17699-14-8	1355	1351	Terpenes	0.04 ± 0.01	0.09 ± 0.01
60	26.09	Copaene	C_15_H_24_	3856-25-5	1380	1376	Terpenes	0.3 ± 0.08	0.48 ± 0.12
61	26.50	(+)-epi-Bicyclosesquiphellandrene	C_15_H_24_	54274-73-6	1392	\	Terpenes	0.05 ± 0.01	0.09 ± 0.06
62	27.24	Santalene	C_15_H_24_	512-61-8	1424	1420	Terpenes	0.03 ± 0.01	0.05 ± 0.02
63	28.07	*γ*-Muurolene	C_15_H_24_	30021-74-0	1466	1477	Terpenes	0.01 ± 0.01	0.03 ± 0.01
64	28.47	Germacrene D	C_15_H_24_	23986-74-5	1486	1481	Terpenes	0.04 ± 0.02	0.05 ± 0.03
65	28.79	(R)-*β*-himachalene	C_15_H_24_	1461-03-6	1502	1500	Terpenes	0.01 ± 0.01	0.03 ± 0.01
66	29.17	(+)-δ-Cadinene	C_15_H_24_	483-76-1	1529	1524	Terpenes	0.34 ± 0.1	0.65 ± 0.26
67	29.47	*α*-Calacorene	C_15_H_20_	21391-99-1	1550	1542	Others	0.02 ± 0.01	0.03 ± 0.01
		Terpenes						72.46	46.35
		Alcohols						18.30	38.33
		Aromatic						2.94	3.99
		Ketones						2.32	4.62
		Aldehydes						0.94	1.26
		Esters						0.82	1.85
		Others						0.42	0.87
		Acids						0.00	0.20
		Total identified compounds						98.20	97.46

^a^ RT: Retention time; ^b^ RI: Retention index on the HP-5MS column. ^c^ RI_ref_: Retention indices were obtained from the NIST Mass Spectral Library (June 2017) and the Wiley Registry of Mass Spectral Data, 8th Edition. ^d^ Relative content (%) = (area under peak/total area) × 100. The relative content was calculated using the area normalization method and expressed as mean ± SD (n = 3); “-”: Not detected.

## Data Availability

The original contributions presented in this study are included in the article/Supplementary Materials. Further inquiries can be directed to the corresponding authors.

## Supplementary Materials

The following supporting information can be downloaded at: https://www.mdpi.com/article/10.3390/molecules30071404/s1, Figure S1. Total ion chromatograms (TICs) for the QC sample in positive ion modes (ESI^+^) and negative ion modes (ESI^−^); Figure S2. The fragmentation behavior of (A) phenylethanoid glycosides and (B) forsythenside B in negative ion mode; Figure S3. The MS/MS spectra of ursolic acid in ESI^+^ and ESI^−^ (A). Molecular network analysis with CD software identified 30 triterpenoid compounds in ESI^+^ mode; Figure S4. 200 times permutation test plots: (A) ESI^+^ mode with 798 features and (B) ESI^−^ mode with 1071 features; Figure S5. Heatmap of 43 differential non-volatile compounds; Figure S6. Hierarchical Clustering Analysis (HCA) based on the differential metabolites screened by LC-MS (A) and HS-GC-MS (B); Table S1. The detailed information of 237 compounds identified through LC-MS analysis; Table S2. The detailed information of all 29 reference standards used in this study; Table S3. The detailed information of Forsythiae Fructus samples; Table S4. Differential compounds identified through LC-MS and HS-GC-MS analysis; Table S5. Performance and validation results of OPLS-DA models for GFF and RFF classification based on single techniques and mid-level data fusion.
