# Supplementary material for: Mid-Level Data Fusion Techniques of LC-MS and HS-GC-MS for Distinguishing Green and Ripe Forsythiae Fructus
Source: Molecules. 2025 Mar 21;30(7):1404. doi: 10.3390/molecules30071404 (PMC11990718; doi:10.3390/molecules30071404)
Supplement: Supplementary file 1 [file molecules-30-01404-s001.zip › molecules-3493732-supplementary.pdf]

Table S1. The detailed information of 237 compounds identified through LC-MS analysis.

NO	RT (min)	Adduct	Formula	*m/z*	Error (ppm)	MS/MS	Identification or characterization	Classification
1	0.98	$[M-H]^-$	$C_7H_{12}O_6$	191.0560	-0.74	173.0463, 127.0405	Quinic acid	Organic acids
2	1.04	$[M+H]^+$	$C_9H_{11}NO$	150.0914	0.14	132.0810, 118.0653	Indolin-2-ylmethanol	Alkaloids
3	1.50	$[M-H]^-$	$C_{14}H_{24}O_9$	335.1346	-0.35	179.0575, 155.0719, 119.0353, 113.0247, 101.0246, 89.0246, 85.0297	cis-1-(*β*-D-Glucopyranosyloxy)-4-hydroxycyclohexaneacetic acid	Cyclohexyl ethanol derivatives
4	1.51	$[M-H]^-$	$C_{14}H_{26}O_8$	321.1554	-3.68	161.0464, 101.0250	Rengyoside A	Cyclohexyl ethanol derivatives
5	1.56	$[M-H]^-$	$C_{14}H_{20}O_8$	315.1085	-0.41	**153.0559**, **135.0457**, 89.0246	2-(3,4-Dihydroxyphenyl) ethyl *β*-D-glucopyranoside	Cyclohexyl ethanol derivatives
6	1.62	$[M-H]^-$	$C_8H_{14}O_4$	173.0818	-0.66	113.0607	cis-1,4-Dihydroxycyclohexaneacetic acid	Cyclohexyl ethanol derivatives
7	1.94	$[M+H]^+$	$C_9H_{11}NO$	150.0914	0.05	132.0806, 118.0650, 100.0243	Forsyqinlingine C	Alkaloids
8[#]	2.52	$[M-H]^-$	$C_{14}H_{20}O_8$	315.1084	-1.85	**153.0571**, **135.0455**, 119.0350, 113.0244, 108.0219, 101.0244, 89.0246	Cornoside	Cyclohexyl ethanol derivatives
9	2.81	$[M-H]^-$	$C_8H_8O_4$	167.0349	-0.55	123.0457, 108.0220	Vanillic acid	Organic acids
10	3.03	$[M+H]^+$	$C_8H_{10}O_2$	139.0754	0.38	121.0650, 111.0806, 97.0648, 79.0542	Tyrosol	Others
11	3.07	$[M-H]^-$	$C_{14}H_{24}O_8$	319.1397	-2.89	161.0452, 157.0872, 119.0355, 113.0249, 101.0245, 73.0299	Rengyoside B	Cyclohexyl ethanol derivatives
12	3.46	$[M-H]^-$	$C_{14}H_{24}O_9$	335.1346	-0.34	179.0579, 161.0463, 131.0347, 119.0352, 113.0246, 101.0246	Rengynic acid-1-*O*-*β*-D-glucopyranoside	Cyclohexyl ethanol derivatives
13	3.92	$[M-H]^-$	$C_{14}H_{20}O_8$	315.1085	-0.28	153.0554, 135.0459, 119.0353,	Vanilloloside	Organic acids
14	4.13	$[M-H]^-$	$C_{13}H_{16}O_9$	315.0721	-0.24	153.0191, 109.0298	1,5-Anhydro-6-*O*-(3,4,5-trihydroxybenzoyl)-D-glucitol	Organic acids

15	4.74	[M−H]$^-$	$C_8H_{10}O_3$	153.0557	-0.32	123.0454, 93.0351	Hydroxytyrosol	Organic acids
16	5.29	[M−H]$^-$	$C_{14}H_{20}O_8$	315.1085	-0.24	**153.0560**, **135.0456**, 119.0351, 113.0244, 101.0247, 89.0246	2-(3,4-Dihydroxyphenyl) ethyl β-D-glucopyranoside	PhGs
17	5.49	[M−H]$^-$	$C_{20}H_{30}O_{13}$	477.1605	-1.71	449.4005, 315.1103, 179.0560, 161.0463, **135.0455**, 119.0354, 113.0253, 101.0244, 89.0249,	Forsythoside D	PhGs
18#	6.01	[M−H]$^-$	$C_{16}H_{24}O_{10}$	375.1284	-3.20	337.1743, 213.0771, 169.0872, 151.0768, 125.0611, 121.0661, 119.0355, 113.0252, 101.0248	Adoxosidic acid	Iridoids
19	6.15	[M−H]$^-$	$C_7H_6O_3$	137.0244	-0.25	109.0305, 93.0348	4-Hydroxybenzoic acid	Organic acids
20	6.28	[M−H]$^-$	$C_{16}H_{22}O_{11}$	389.1077	-3.05	345.1214, 183.0665, 165.0560, 121.0662, 119.0355, 113.0249, 101.0246	Forsythide	Iridoids
21#	6.66	[M−H]$^-$	$C_{14}H_{20}O_7$	299.1135	-2.30	119.0350, 101.0248	Salidroside	PhGs
22	6.76	[M−H]$^-$	$C_{19}H_{28}O_{12}$	447.1499	-2.07	315.1107, 191.0566, 149.0456, **135.0450**, 131.0353, 123.0455, 113.0253, 101.0248	Rebouoside B	PhGs
23	7.00	[M−H]$^-$	$C_{22}H_{28}O_{12}$	483.1500	-1.12	461.1671, 337.0872, 315.111, 205.0721, 163.0623, **153.0557**, 151.3092, **135.0456**, 131.0350, 123.0453, 115.0404, 101.0244	Unknown	PhGs
24#	7.19	[M−H]$^-$	$C_{20}H_{30}O_{12}$	461.1655	-2.05	315.1081, 205.0720, 163.0613, **153.0561**, **135.0454**, 131.0353, 123.0454, 119.0355, 101.0247	Forsythoside E	PhGs
25	7.31	[M−H]$^-$	$C_{21}H_{28}O_{13}$	487.1450	-1.52	221.0460, **179.0352**, 161.0246, 149.0608, 147.0461, **135.0453**, 133.0297, 131.0356, 101.0246	93675-89-9	PhGs

26	7.56	$[M-H]^-$	$C_{19}H_{28}O_{11}$	431.1550	-2.07	191.0564, 149.0457, 131.0349, 121.9629, 101.0249	Benzyl *β*-D-cellobioside	Organic acids
27	7.69	$[M-H]^-$	$C_9H_{10}O_4$	181.0505	-0.49	137.0609, 121.0300, 59.0139	(2S)-2,3-Dihydroxy-1-(4-hydroxyphenyl)-1-propanon	Others
28	7.76	$[M-H]^-$	$C_{19}H_{28}O_{11}$	431.1550	-1.99	299.1140, 191.0568, 149.0460, 137.0603, 131.0353, 119.0504, 113.0251, 101.0245	Darendoside A	PhGs
29	7.78	$[M-H]^-$	$C_{16}H_{18}O_9$	353.0866	-3.55	191.0564, 93.0351	Chlorogenic acid	Organic acids
30	7.80	$[M-H]^-$	$C_{22}H_{28}O_{11}$	467.1556	-0.41	151.0433, 149.0247, 135.0452, 113.0248, 108.0219, 101.0248	6-*O*-p-Hydroxybenzoyl ajugol	Iridoids
31	7.82	$[M-H]^-$	$C_{21}H_{22}O_{12}$	465.1040	0.38	149.0246, 135.0459, 121.0298, 108.0218, 101.0248	Plantagoside	Flavonoids
32[#]	7.86	$[M-H]^-$	$C_{22}H_{26}O_{11}$	465.1396	-1.15	161.0456, 153.0552, 149.0247, **135.0454**, 121.0297, 101.0245	Forsythenside B	Cyclohexyl ethanol derivatives
33	8.17	$[M-H]^-$	$C_9H_8O_4$	179.0349	-0.43	179.0352, 135.0454, 117.0350, 91.0558	Caffeic acid	Organic acids
34	8.88	$[M-H]^-$	$C_{21}H_{28}O_{13}$	487.1450	-1.41	163.0403, 145.0297, 119.0504,117.0348	Swertiamacroside	PhGs
35	8.92	$[M-H]^-$	$C_{29}H_{36}O_{17}$	655.1878	-0.33	637.1765, 545.1532, 493.1566, 475.1477, 457.1358, 443.1217, 383.0961, 193.0509, **179.0351**, **161.0245**, 151.0402, **135.0453**,	Hellicoside	PhGs
36	9.31	$[M+H]^+$	$C_{17}H_{25}NO$	260.2009	-0.17	245.1776, 230.1544, 228.1746, 172.1120, 160.1115, 146.0964	Forsyshiyanine A	Alkaloids
37	9.33	$[M-H]^-$	$C_{16}H_{18}O_8$	337.0929	-0.05	191.0565, 173.0457, 163.0402, 145.0300, 119.0505, 93.0347	4-*O*-*p*-Coumaroylquinic acid	Organic acids
38	9.39	$[M-H]^-$	$C_{22}H_{30}O_{11}$	469.1709	-1.42	193.0505, 161.0617, 151.0403, 133.0296, 123.0459, 106.0428	Rengyoside D	Cyclohexyl ethanol derivatives

39	9.45	[M−H]⁻	$C_{22}H_{28}O_{11}$	467.1552	-1.49	193.0507, 151.0405, 135.0454, 133.0292, 113.0246, 108.0218, 101.0247	6-*O*-*p*-Hydroxybenzoyl ajugol	Iridoids
40	9.60	[M−H]⁻	$C_{29}H_{36}O_{16}$	639.1928	-0.39	621.1878, **179.0353**, **161.0246**, 151.0404, **135.0455**	*β*-Hydroxyacteoside	PhGs
41	9.65	[M−H]⁻	$C_{18}H_{22}O_{11}$	413.1079	-2.40	267.0724, 249.0617, 231.0515, 163.0401, 145.0298, 119.0504	Unkown	Others
42	9.80	[M−H]⁻	$C_{28}H_{34}O_{16}$	625.1771	-0.42	607.1711, 455.1209, 445.1333, 221.0468, **179.0352**, **161.0247**, 151.0402, 149.0461, **135.0455**, 133.0299, 131.0356	935509-31-2	PhGs
43	9.91	[M−H]⁻	$C_{27}H_{34}O_{14}$	581.1875	-0.32	623.2003, 461.1664, 443.1569, 351.5221, **179.0349**, **161.0246**, 137.0246, **135.0455**, 133.0297	2-(4-Hydroxyphenyl) ethyl 2-*O*-[(2S,3R,4S)-3,4-dihydroxy-4-{[(4-hydroxy-3-methoxybenzoyl) oxy] methyl} tetrahydro-2-furanyl]-beta-D-glucopyranoside	PhGs
44	9.93	[M−H]⁻	$C_{26}H_{32}O_{12}$	535.1817	-0.49	**373.1309**, 193.0506, 179.0720, 163.0409, **151.0405**, 146.0376	(+)-8-Hydroxypinoresinol 4-*O*-*β*-D-glucopyranoside	Lignans
45	10.00	[M−H]⁻	$C_{22}H_{26}O_{10}$	449.1445	-1.83	151.0406, **135.0458**, 133.0300, 107.0506	Forsythenside A	Cyclohexyl ethanol derivatives
46	10.05	[M−H]⁻	$C_{17}H_{24}O_{11}$	403.1236	-2.53	371.0999, 327.1099, 191.0359, 179.0715, 147.0452, 139.0403, 137.0610, 127.0410, 119.0507, 101.0246	Hastatoside	Iridoids
47*	10.08	[M−H]⁻	$C_{32}H_{32}O_{18}$	703.1547	-4.39	557.0991, 541.1245, 523.1126, 395.0653, 377.0552, 275.0234, **179.0354**, 161.0248, **153.0565**, 151.0402, **135.0455**, 133.0296	Unknown	PhGs

48	10.18	[M−H]⁻	$C_{29}H_{36}O_{16}$	639.1927	-0.53	477.1619, 459.1502, 315.1108, 221.0672, **179.0352**, **161.0246**, **153.0561**, **135.0454**, 123.0454, 119.0350, 113.0244, 105.0348, 101.0244	Plantamajoside	PhGs
49*	10.18	[M−H]⁻	$C_{31}H_{34}O_{16}$	661.1746	-4.11	499.1429, 481.1300, 459.1508, 431.4624, 337.0915, 319.0799, 283.0602, 235.0595, 193.0494, **179.0353**, 163.0393, **161.0247**, **153.0560**, **135.0454**, 125.0245, 113.0246, 101.0245	Unknown	PhGs
50*	10.24	[M−H]⁻	$C_{30}H_{32}O_{14}$	615.1718	-4.00	499.1429, 481.1346, 459.1506, 337.0914, 319.0802, 301.0696, 285.0713, 235.0592, 283.0601, 235.0592, 217.0482, 207.0281, 193.0486, **179.0352**, 161.0246, **153.0559**, 149.0239, **135.0452**, 125.0245, 123.0453, 101.0246	Unknown	PhGs
51	10.30	[M−H]⁻	$C_{32}H_{42}O_{16}$	681.2397	-0.23	357.1336, **151.0404**, 137.0224, 136.0168, 108.0220	Pinoresinol diglucoside	Lignans
52	10.36	[M−H]⁻	$C_{29}H_{36}O_{16}$	639.1928	-0.48	621.1827, 441.1393, **179.0352**, 163.0602, **161.0246**, **135.0453**,	Forsythoside C	PhGs
53	10.37	[M−H]⁻	$C_{31}H_{34}O_{16}$	661.1748	-3.97	643.1657, 481.1278, 463.1181, 441.1412, 335.0772, 317.0661, 215.0330, **179.0351**, 163.0418, **161.0245**, 151.0404, **135.0454**, 133.0296, 119.0507	Unknown	PhGs

54	10.38	$[M+H]^+$	$C_{23}H_{24}O_{11}$	477.1391	-0.08	431.0076, 163.0394, 145.0291, **135.0441**, 123.0445, 117.0340	Forsyoxaside F	PhGs
55	10.40	$[M-H]^-$	$C_{29}H_{36}O_{16}$	639.1928	-0.48	621.1830, 459.1492, 441.1434, **179.0351**, **161.0245**, 151.0401, **135.0452**	*β*-Hydroxyacteoside	PhGs
56	10.57	$[M-H]^-$	$C_{22}H_{26}O_{10}$	449.1444	-1.84	315.1072, 205.0511, 193.0510, 165.0559, 161.0613, 151.0402, **135.0454**, 133.0297	Forsythoside M	PhGs
57	10.80	$[M-H]^-$	$C_{22}H_{28}O_{10}$	451.1601	-1.73	161.0462, 151.0403, 123.0449, 119.0355, 113.0253, 101.0247	[(1S,4aR,5S,7aS)-1-(*β*-D-Glucopyranosyloxy)-5-hydroxy-1,3,4,4a,5,7a-hexahydrocyclopenta[c]pyran-7-yl] methyl benzoate	Iridoids
58	10.80	$[M-H]^-$	$C_{19}H_{28}O_{10}$	415.1600	-2.01	191.0573, 149.0458, 131.0350,	Phenethyl *β*-primeveroside	PhGs
59	10.86	$[M-H]^-$	$C_{29}H_{36}O_{16}$	639.1928	-0.39	477.1613, 459.1545, 315.1091, **179.0351**, **161.0246**, **135.0453**, 133.0297, 123.0451, 113.0245, 101.0247	Suspensaside	PhGs
60	10.89	$[M-H]^-$	$C_{23}H_{26}O_{11}$	477.1395	-1.61	**179.0354**, **161.0250**, **135.0456**, 133.0298	Calceolarioside A	PhGs
61	10.98	$[M-H]^-$	$C_{29}H_{34}O_{16}$	637.1772	-0.36	**179.0355**, **161.0247**, 151.0401, **135.0455**, 133.0297	935509-36-7	PhGs
62	11.02	$[M+H]^+$	$C_{17}H_{25}NO$	260.2009	-0.16	228.1746, 172.1116	Forsyqinlingine A	Alkaloids
63	11.09	$[M-H]^-$	$C_{23}H_{26}O_{11}$	477.1395	-1.46	315.1088, **179.0351**, **161.0245**, **135.0453**, 133.0297, 113.0249	Plantainoside B	PhGs
64	11.11	$[M-H]^-$	$C_{28}H_{34}O_{15}$	609.1822	-0.54	447.1512, 429.1404, **179.0352**, **161.0245**, **135.0453**, 133.0297, 131.0359	Forsythoside J	PhGs
65	11.19	$[M-H]^-$	$C_{29}H_{36}O_{15}$	623.1978	-0.53	461.1687, **179.0355**, **161.0246**, **135.0456**, 133.0296	Forsythoside H	PhGs

66	11.24	$[M-H]^-$	$C_{29}H_{34}O_{16}$	637.1772	-0.37	475.1455, 457.1342, 335.1669, **179.0362**, 167.0356, **161.0246**, 151.0406, 149.0246, 137.0250, **135.0454**	1809835-79-7	PhGs
67	11.25	$[M-H]^-$	$C_{30}H_{38}O_{17}$	669.2034	-0.36	507.1706, 492.1494, 489.1601, **179.0360**, **161.0246**, **135.0451**, 133.0296, 101.0242	Forsythoside C+CH_3OH	PhGs
68	11.27	$[M-H]^-$	$C_{26}H_{32}O_{11}$	535.1816	-0.66	**373.1295**, 343.1180, **313.1063**, 269.0820, 179.0718, 163.0406, **151.0397**, 109.0295	(+)-8-Hydroxypinoresinol 4-*O*-*β*-D-glucopyranoside	Lignans
69	11.40	$[M-H]^-$	$C_{26}H_{34}O_{11}$	521.2024	-0.75	329.1400, 161.0608, 147.0455, 131.0507, 121.0296, 93.0350	Lariciresinol 4-*O*-*β*-D-glucopyranoside	Lignans
70	11.44	$[M-H]^-$	$C_{29}H_{36}O_{16}$	639.1928	-0.34	**179.0359**, **161.0245**, **151.0401**, **135.0452**, 133.0298	Plantamajoside	PhGs
71	11.47	$[M-H]^-$	$C_{27}H_{36}O_{12}$	551.2130	-0.64	359.1494, 191.0721, 163.0404	Forsythiayanoside B	Lignans
72	11.61	$[M-H]^-$	$C_{26}H_{34}O_{11}$	521.2023	-0.74	359.1492, 341.1393, **313.1083**, 113.0243, 109.0297, 101.0246	Glochidioboside	Lignans
73	11.66	$[M-H]^-$	$C_{16}H_{24}O_9$	359.1335	-3.40	197.0818, 153.0923, 135.0818, 113.0249, 101.0250	(2S)-2-(3,4-Dimethoxyphenyl)-2-hydroxyethyl beta-L-glucopyranoside	Others
74	11.69	$[M-H]^-$	$C_{26}H_{32}O_{11}$	581.1874	-0.31	**373.1261**, 343.1176, 163.0407	Forsythialanside E	Lignans
75[#]	11.70	$[M-H]^-$	$C_{29}H_{36}O_{15}$	623.1978	-0.60	461.1656, 443.1554, **179.0350**, **161.0245**, **153.0559**, **135.0454**,	Forsythoside I	PhGs
76	11.72	$[M-H]^-$	$C_{28}H_{34}O_{15}$	609.1821	-0.69	447.1499, 429.1432, 301.0350, 300.0279, 271.0252, 255.0302, 243.0300, 227.0352, 215.0354, 199.0400, **179.0352**, **161.0246**, 151.0035, **135.0451**, 133.0297, 131.0350	Forsythoside J	PhGs

77	11.97	$[M-H]^-$	$C_{28}H_{34}O_{15}$	609.1819	-1.01	447.1505, 429.1407, 315.1081, 271.0250, 255.0295, 227.0352, **179.0351**, 163.0040, **161.0245**, **153.0568**, **135.0453**, 133.0296, 131.0351	Calceolarioside C	PhGs
78*	11.97	$[M-H]^-$	$C_{30}H_{32}O_{15}$	631.1640	-4.49	469.1335, 451.1226, 429.1401, 319.0799, 235.0588, 193.0486, **179.0352**, 161.0246, **153.0559**, **135.0454**, 133.0297, 125.0244, 123.0453, 101.0247	Unknown	PhGs
79#	12.06	$[M-H]^-$	$C_{27}H_{30}O_{16}$	609.1462	0.15	343.0445, 301.0349, 300.0278, 271.0251, 255.0302, 245.0439, 243.0300, 227.0350, 215.0350, 163.0037, 161.0244, **151.0039**, **135.0087**, 133.0294	Rutin	Flavonoids
80	12.25	$[M-H]^-$	$C_{32}H_{46}O_{19}$	733.2559	-0.24	571.2050, 553.1959, 527.2191, 445.1348, 375.1292, 357.1193, 283.0833, 239.0935, 213.0771, 179.0566, 169.0870, 161.0469,	Unknown	Others
81	12.27	$[M-H]^-$	$C_{21}H_{22}O_9$	417.1182	-2.29	255.0665, 153.0195, **135.0089**, 119.0504	Liquiritin	Flavonoids
82#	12.36	$[M-H]^-$	$C_{29}H_{36}O_{15}$	623.1979	-0.39	461.1692, 443.1567, **179.0353**, **161.0246**, **135.0454**, 133.0297	Isoforsythiaside	PhGs
83	12.46	$[M-H]^-$	$C_{26}H_{30}O_{13}$	549.1609	-0.84	255.0664, 153.0198, 135.0087,	Licuraside	Others
84	12.50	$[M-H]^-$	$C_{28}H_{34}O_{15}$	609.1820	-0.87	447.1505, 271.0265, **179.0361**, **161.0246**, **135.0454**, 133.0297	Lianqiaoxinoside C	PhGs
85	12.54	$[M-H]^-$	$C_{26}H_{34}O_{11}$	521.2024	-0.90	329.1399, 175.0767, 161.0611, 159.0288, 136.0528, 131.0507	Lariciresinol 4-*O*-*β*-D-glucopyranoside	Lignans

86	12.67	$[M-H]^-$	$C_{30}H_{38}O_{16}$	653.2085	-0.33	621.1834, 487.1478, 459.1528, 427.1233, 179.0354, 163.0618, 161.0246, 151.0404, 149.0609, **135.0454**, 133.0298, 109.0296, 101.0246	S-Suspensaside methyl ether	Lignans
87*	12.67	$[M-H]^-$	$C_{42}H_{32}O_{13}$	743.1776	0.75	643.1649, 441.1407, 243.0286, **179.0355**, 161.0243, 151.0406, **135.0457**	Unknown	PhGs
88*	12.67	$[M-H]^-$	$C_{32}H_{36}O_{16}$	675.1903	-4.03	643.1660, 497.1090, 441.1400, 243.0275, 215.0329, 193.0489, **179.0353**, 163.0403, 161.0244, **153.0558**, 151.0406, 149.0249, **135.0454**, 131.0115, 119.0506	Unknown	PhGs
89	12.93	$[M-H]^-$	$C_{23}H_{26}O_{11}$	477.1395	-1.44	315.1104, **179.0346**, **161.0247**, **135.0455**, 133.0298	Plantainoside A	PhGs
90#	13.00	$[M-H]^-$	$C_{34}H_{44}O_{19}$	755.2402	-0.34	593.2101, 447.1499, **179.0373**, **161.0246**, **153.0560**, **135.0453**, 133.0296	Forsythoside B	PhGs
91*	13.35	$[M-H]^-$	$C_{31}H_{34}O_{15}$	645.1797	-4.25	465.1366, 443.1558, 205.0718, **179.0353**, 161.0247, **153.058**, **135.0453**	Unknown	PhGs
92	13.41	$[M-H]^-$	$C_{30}H_{36}O_{16}$	651.1928	-0.39	427.1253, 181.0508, 179.0351, 163.0611, 161.0246, 149.0246, 135.0453, 133.0297, 131.0350, 121.0296, 109.0295	Adoxosidic acid-6'-oleuroperic ester	Iridoids
93#	13.53	$[M-H]^-$	$C_{29}H_{36}O_{15}$	623.1978	-0.53	461.1673, 443.1575, **179.0354**, **161.0246**, **153.0550**, **135.0453**, 133.0297, 131.0351	Forsythoside I	PhGs
94#	13.54	$[M-H]^-$	$C_{29}H_{36}O_{15}$	623.1978	-0.50	461.1658, 443.1563, 315.1178, **179.0352**, **161.0245**, **135.0453**,	Forsythoside A	PhGs

95	13.87	[M−H]$^-$	$C_{37}H_{50}O_{18}$	781.2924	-0.05	621.1847, 427.1216, **179.0354**, 161.0246, **135.0452**, 133.0297	Unknown	PhGs
96#	14.15	[M−H]$^-$	$C_{29}H_{36}O_{15}$	623.1978	-0.50	461.1663, 443.1549, 315.1077, **179.0348**, **161.0245**, **153.0556**, **135.0453**, 133.0296, 123.0453, 117.0345, 113.0244, 101.0241	Acteoside	PhGs
97	14.20	[M−H]$^-$	$C_{24}H_{34}O_{10}$	481.2073	-0.87	315.1106, 225.1130, 197.1186, 183.1026, **135.0456**	Forsythenside I	Cyclohexyl ethanol derivatives
98#	14.21	[M−H]$^-$	$C_{23}H_{26}O_{11}$	477.1395	-1.47	315.1085, 281.0664, 221.0439, **179.0352**, **161.0246**, **135.0452**, 133.0297, 123.0454, 117.0352,	Calceolarioside B	PhGs
99*	14.36	[M−H]$^-$	$C_{31}H_{40}O_{17}$	683.2190	-0.40	651.1942, 434.7393, **179.0356**, 161.0246, **135.0453**, 133.0298	Unknown	PhGs
100	14.62	[M−H]$^-$	$C_{24}H_{28}O_{11}$	491.1553	-1.28	345.4548, 235.0624, 193.0516, 175.0400, 161.0225, 160.0168, **135.0440**	Forsythenside J	Cyclohexyl ethanol derivatives
101	14.85	[M−H]$^-$	$C_{28}H_{34}O_{14}$	593.1873	-0.52	447.1539, 429.1393, 285.0403, 255.0301, 227.0353, 163.0404, 145.0302, 119.0501	*β*-(4-hydroxyphenyl) ethyl-4-O-E-caffeoyl-O-[*β*-D-apiofuranosyl-(1->2)]-*β*-D-glucopyranoside	PhGs
102	14.95	[M−H]$^-$	$C_{27}H_{30}O_{15}$	593.1511	-0.12	447.1546, 327.0497, 285.0403, 255.0300, 227.0350, 163.0035, **151.0037**, **135.0085**, 117.0347, 107.0141	Kaempferol 3-*O*-rutinoside	Flavonoids
103	15.12	[M−H]$^-$	$C_{20}H_{28}O_{11}$	443.1551	-1.86	427.8166, **149.0611**	Forsythenside L	Cyclohexyl ethanol derivatives
104#	15.42	[M−H]$^-$	$C_{26}H_{32}O_{11}$	519.1867	-0.98	357.1334, 342.1089, 311.1313, 163.0404, 161.0261, 151.0402, 136.0166, 122.0379, 121.0296, 113.0250	(+)-Pinoresinol 4′-*O*-*β*-D-glucopyranoside	Lignans

105	15.98	$[M+H]^+$	$C_{18}H_{17}NO_3$	296.1281	-0.10	132.0807, 117.0573	Forsyshiyanine B	Alkaloids
106	16.00	$[M-H]^-$	$C_{29}H_{34}O_{15}$	621.1822	-0.53	459.1518, 441.1408, 179.0351, 161.0246, 151.0402, 149.0245, **135.0453**, 133.0297, 101.0245	Suspensaside A	Lignans
107	16.19	$[M-H]^-$	$C_{10}H_{10}O_4$	193.0505	-0.64	161.0246, 133.0297, 95.6540	*trans*-Caffeic acid methyl ester	Others
108*	16.50	$[M-H]^-$	$C_{41}H_{30}O_{12}$	713.1674	1.34	667.1462, 645.1810, 483.1478, 465.1369, 443.1556, 193.0502, **179.0350**, 161.0245, **153.0559**, **135.0453**, 133.0295, 101.0247	Unknown	PhGs
109*	16.50	$[M-H]^-$	$C_{31}H_{34}O_{15}$	645.1798	-4.08	483.1497, 465.1403, 443.1559, 337.0929, 319.0801, **179.0354**, 161.0248, **153.0559**, **135.0455**, 133.0299, 101.0247	Unknown	PhGs
110#	16.51	$[M-H]^-$	$C_{29}H_{36}O_{15}$	623.1978	-0.48	461.1663, 443.1546, **179.0351**, **161.0245**, **153.0553**, **135.0453**,	Isoacteoside	PhGs
111	16.89	$[M-H]^-$	$C_{30}H_{38}O_{16}$	653.2084	-0.41	473.1648, 458.1394, **179.0351**, **161.0244**, **135.0453**, 133.0295, 131.0352, 101.0243	Campneoside I	PhGs
112	17.68	$[M-H]^-$	$C_{26}H_{32}O_{11}$	519.1867	-0.45	357.1334, 342.1100, 163.0412, 151.0401	Koreanaside A	Lignans
113	17.99	$[M-H]^-$	$C_{29}H_{36}O_{14}$	607.2030	-0.31	461.1656, 443.1556, 297.0985, 163.0402, **153.0566**, 145.0296, **135.0453**, 131.0360, 119.0503, 101.0248	Forsythenside K	PhGs
114	18.20	$[M-H]^-$	$C_{20}H_{22}O_7$	373.1282	-2.97	358.1038, **313.1088**, 163.0402, 108.0217	(+)-1-Hydroxy-6-epipinoresinol	Lignans
115	18.49	$[M-H]^-$	$C_{22}H_{26}O_9$	433.1496	-1.90	193.0499, 179.0561, 175.0402, 151.0400, 133.0297, 123.0453, 119.0348, 113.0247	3-Hydroxy-4-[3-(4-methoxyphenyl) propanoyl] phenyl beta-D-glucopyranoside	Others

116	18.58	[M−H]⁻	$C_{20}H_{22}O_7$	373.1282	-2.99	358.1038, **313.1079**, 163.0403, 108.0219	8-Hydroxypinoresinol	Lignans
117	18.71	[M−H]⁻	$C_{26}H_{32}O_{11}$	519.1870	-0.43	487.9436, 357.1336, 179.0353, 151.0403	4-[4-(4-Hydroxy-3-methoxyphenyl) tetrahydro-1H,3H-furo[3,4-c] furan-1-yl]-2-methoxyphenyl hexopyranoside	Lignans
118	18.73	[M−H]⁻	$C_{27}H_{34}O_{13}$	565.1925	-0.37	357.1336, 342.1100, 327.1258, 163.0412, 151.0403, 137.0243, 135.0451, 109.0301	Fraxiresinol-4′-*O*-*β*-D-glucopyranoside	Lignans
119	19.84	[M−H]⁻	$C_{26}H_{32}O_{11}$	519.1868	-0.69	357.1335, 342.1098, 137.0614, 122.0375	(+)-Epipinoresinol-*β*-D-glucoside	Lignans
120	20.29	[M−H]⁻	$C_{24}H_{34}O_{10}$	481.2074	-0.75	179.1083, **135.0458**, 119.0352, 101.0244, 89.0247	Forsythenside G	Cyclohexyl ethanol derivatives
121	20.40	[M−H]⁻	$C_{26}H_{32}O_{11}$	519.1867	-0.91	357.1335, 342.1098, 137.0614, 122.0375	Matairesinoside	Lignans
122	21.47	[M−H]⁻	$C_{12}H_{20}O_5$	243.1237	-0.30	225.1138, 207.1030	1,4-Cyclohexanediol, 1-[2-(acetyloxy) ethyl]-, 4-acetate, cis	Cyclohexyl ethanol derivatives
123	21.97	[M−H]⁻	$C_{15}H_{12}O_4$	255.0662	-0.20	153.0195, **135.0090**, 119.0504, 93.0348, 91.0190	Liquiritigenin	Flavonoids
124	22.27	[M−H]⁻	$C_{21}H_{22}O_9$	417.1182	-2.24	255.0660, 153.0202, **135.0088**,	Isoliquiritin	Flavonoids
125	22.50	[M−H]⁻	$C_{48}H_{68}O_{28}$	1091.3822	-0.24	929.3305, 883.8681, 733.2557, 715.2502, 571.2033, 553.1916, 445.1343, 375.1285, 357.1173, 151.0765	Forsydoitriside A	Iridoids
126#	22.72	[M−H]⁻	$C_{21}H_{24}O_6$	579.2081	-0.38	371.1492, 356.1278, 121.0294	Phillygenin	Lignans
127	22.79	[M−H]⁻	$C_{25}H_{30}O_{12}$	521.1660	-0.82	315.1242, 163.0400, 149.0121, 145.0293, 119.0502, 117.0346	Suspenoidside A	Iridoids
128	23.38	[M−H]⁻	$C_{26}H_{32}O_{11}$	519.1868	-0.79	357.1326, 342.1097, 151.0402, 136.0168, 121.0301	Simplocosin	Lignans
129	23.55	[M−H]⁻	$C_{25}H_{30}O_{12}$	521.1660	-0.84	477.1767, 359.1133, 315.1240, 297.1133, 279.9502, 163.0401	Suspenoidside B	Iridoids

130	23.62	[M−H]⁻	$C_{15}H_{10}O_7$	301.0353	-0.12	273.0401, **151.0037**, 107.0139, 93.0348, 65.0033	Quercetin	Flavonoids
132	23.76	[M−H]⁻	$C_{28}H_{36}O_{13}$	579.2081	-0.41	371.1505, 356.1262, 121.0295	Acanthoside B	Lignans
131[#]	23.76	[2M−H]⁻	$C_{27}H_{34}O_{11}$	1067.4106	-0.47	371.1507, 356.1276, 121.0296	Phillyrin	Lignans
133	23.90	[M−H]⁻	$C_{27}H_{44}O_{11}$	543.2807	-0.65	373.3946, 335.2225, 137.0975	Unknown	Others
134	23.99	[M−H]⁻	$C_{26}H_{32}O_{13}$	551.1767	-0.62	345.1339, 327.1238, 193.0508, 179.0366, 149.0614, 133.0295	Suspenoidside E	Iridoids
135[#]	23.99	[M−H]⁻	$C_{27}H_{34}O_{11}$	579.2082	86118.44	371.1490, 356.1264, 136.0533, 121.0296, 83.0138	Arctiin	Lignans
136	24.12	[M−H]⁻	$C_{22}H_{38}O_{10}$	461.2337	-1.01	315.1816, 161.0464, 101.0249	2-(4-Methyl-3-cyclohexen-1-yl)-2-propanyl 6-*O*-(6-deoxy-*α*-L-mannopyranosyl)-*β*-D-glucopyranoside	Others
137	24.32	[M+H]⁺	$C_{20}H_{18}O_4$	323.1278	-0.07	308.1024, 305.1163, 294.0887, 291.1014, 277.0861, 263.1070,	Cyclocumarol	Others
138*	24.36	[M−H]⁻	$C_{48}H_{66}O_{27}$	1073.3712	-0.29	911.3201, 883.3246, 841.3160, 733.2566, 715.2492, 705.2800, 687.2585, 679.2607, 553.1921, 375.1279, 357.1191, 151.0768	Unknown	Others
139	24.48	[M+H]⁺	$C_{21}H_{24}O_7$	389.1596	0.14	335.1281, 151.0752, 151.0385, 107.0492	Forsythialan B	Lignans
140	24.54	[M−H]⁻	$C_{20}H_{34}O_5$	353.2322	-3.23	315.4208, 309.2438, 289.2214, 279.2341	(13E)-11,15-Dihydroxy-9-oxoprost-13-en-1-oic acid	Organic acids
141	25.42	[M−H]⁻	$C_{22}H_{38}O_{10}$	461.2383	-1.10	433.6233, 163.0639, 119.0349, 101.0245	2-(4-Methyl-3-cyclohexen-1-yl)-2-propanyl 6-*O*-(6-deoxy-*α*-L-mannopyranosyl)-*β*-D-glucopyranoside	Others
142	25.45	[M−H]⁻	$C_{35}H_{40}O_{15}$	699.2295	0.04	327.1252, 295.0815, 151.0400, 136.0167, 107.0505	8a-hydroxypinoresinol-4a-*O*-b-(6-p-methoxyphenylacetyl) glucopyranoside	Lignans

143	25.59	$[M-H]^-$	$C_{18}H_{26}O_4$	305.1758	-0.23	261.1860, 243.1758, 191.1450, 107.0504	Unknown	Others
144	25.80	$[M-H]^-$	$C_{20}H_{22}O_6$	357.1332	-3.33	342.1097, **313.1438**, 298.1202, 179.0998, 161.0611, 137.0608, 122.0374, 93.0347	(-)-Matairesinol	Lignans
145	25.80	$[M+H]^+$	$C_{20}H_{22}O_6$	359.1490	0.20	341.1395, 327.2383, 291.1017, 263.1066, 231.0801, 191.0699, 179.0703, 163.0754, 151.0757, 151.0387, 137.0597, 135.0440, 131.0492	rel-(7R,8′R,8R)-Forsythialan C	Lignans
146	25.81	$[M-H]^-$	$C_{25}H_{30}O_{13}$	537.1610	-0.68	243.0665, 137.0250, 135.0089, 109.0298, 108.0219, 91.0191	Suspenoidsides D	Iridoids
147	25.94	$[M-H]^-$	$C_{35}H_{40}O_{14}$	683.2344	0.00	371.1493, 163.0401, 151.0404, 149.0245, 135.0455, 121.0296	Phillyrigeninside B	Lignans
148	25.97	$[M-H]^-$	$C_{36}H_{42}O_{16}$	729.2400	-0.05	371.1492, 357.1299, 163.0409, 149.0246, 135.0452, 121.0297	Unknown	Others
149	26.12	$[M-H]^-$	$C_{20}H_{22}O_6$	357.1332	-3.30	342.1105, 327.1259, 312.1010, 163.0405, 161.0611	Pinoresinol	Lignans
150	26.35	$[M-H]^-$	$C_{20}H_{30}O_5$	349.2009	-3.35	331.1915, 305.2126, 287.2013, 259.2067	3a,14,15,18-Tetrahydroxy-5b,9b-H,10a-labda-8(20),12-dien-16-oic acid γ-lactone	Terpenes
151	26.55	$[M+H]^+$	$C_{20}H_{30}O_3$	319.2266	-0.35	301.2170, 255.2127, 161.1332, 147.1171, 119.0858, 93.0701	11-α-Hydroxy-17-methyltestosterone	Terpenes
152	26.58	$[M-H]^-$	$C_{20}H_{22}O_6$	357.1332	-3.26	342.1123, 327.1236, 312.1001, 163.0407, 161.0606	(+)-Epipinoresinol	Lignans
153	26.70	$[M+H]^+$	$C_{20}H_{32}O_4$	337.2374	0.21	319.2273, 301.2162, 119.0856	Forsyshiyanin B	Lignans

154	26.87	$[M+H]^+$	$C_{20}H_{30}O_4$	335.2217	-0.60	661.3606, 351.0560, 193.0356, 175.0243, 113.0246	7-hydroxy-1,4a-dimethyl-9-oxo-7-(propan-2-yl)-1,2,3,4,4a,4b,5,6,7,9,10,10a-dodecahydrophenanthrene-1-carboxylic acid	Terpenes
155	27.12	$[M-H]^-$	$C_{42}H_{62}O_{17}$	837.3916	0.15	661.3606, 351.0560, 193.0356, 175.0243, 113.0246	LicoricesaponinI G2	Terpenes
156	27.34	$[M+H]^+$	$C_{20}H_{30}O_4$	335.2217	0.02	317.2115, 299.2018, 289.2157, 275.2018, 257.1903, 229.1947, 133.1012, 131.0857, 121.0650	Forsypensin D	Terpenes
157	27.40	$[M+H]^+$	$C_{20}H_{26}O_3$	315.1943	-4.21	269.1905, 241.1952, 193.1225, 147.1176, 119.0860, 95.0489	1,4a-dimethyl-9-oxo-7-(propan-2-yl)-1,2,3,4,4a,9,10,10a-octahydrophenanthrene-1-carboxylic acid	Terpenes
158	27.41	$[M-H]^-$	$C_{32}H_{50}O_{14}$	657.3127	-0.12	537.2722, 375.2170, 315.1973, 179.0560, 161.0465, 113.0244, 101.0244, 89.0246	(2*α*,3*β*,13*α*)-2,19-Dihydroxy-7-oxopimara-8,15-dien-3-yl 6-*O*-*α*-D-glucopyranosyl-*β*-D-altropyranoside	Terpenes
159	27.51	$[M+H]^+$	$C_{15}H_{12}O_4$	257.0809	0.04	239.0701, 211.0758, 147.0440, 137.0232, 119.0490, 91.0544, 81.0335	Isoliquiritigenin	Flavonoids
160	27.52	$[M+H]^+$	$C_{20}H_{28}O_5$	349.2009	-0.17	285.1856, 267.1732, 191.1433, 163.0745, 135.0804, 119.0856, 107.0857, 95.0491, 93.0701	Forsypensin E	Terpenes
161	27.58	$[M-H]^-$	$C_{20}H_{30}O_5$	349.2009	-3.27	331.1919, 305.2123, 287.2019	Unknown	Others
162	27.63	$[M-H]^-$	$C_{16}H_{12}O_4$	267.0663	-0.09	252.0427, 224.0468, **135.0090**, 91.0189	Formononetin	Flavonoids
163[#]	27.73	$[M-H]^-$	$C_{21}H_{24}O_6$	371.1488	-2.96	151.0764, 136.0529, 121.0295, 93.0345	(-)-Arctigenin	Lignans

164	27.78	$[M+H]^+$	$C_{20}H_{30}O_4$	335.2217	0.03	317.2120, 299.2019, 163.0761, 135.0804, 107.0855, 93.0700, 81.0703	2093050-64-5	Terpenes
165	27.98	$[M+H]^+$	$C_{20}H_{30}O_4$	335.2217	0.01	317.2104, 299.1995, 81.0701	51014-28-9	Terpenes
166	28.10	$[M-H]^-$	$C_{42}H_{62}O_{17}$	837.3915	0.14	661.3612, 351.0559, 193.0355, 113.0245	Licoricesaponin G2	Terpenes
167	28.26	$[M+H]^+$	$C_{20}H_{28}O_5$	3349.2009	0.18	285.1837, 187.1490, 95.0492	Forsypensin E	Terpenes
168	28.49	$[M+H]^+$	$C_{20}H_{30}O_5$	351.2166	-0.01	333.2062, 319.4277, 161.0962, 145.1017, 121.0650, 81.0699	Forsypensin C	Terpenes
169	28.49	$[M+H]^+$	$C_{20}H_{30}O_5$	333.2060	-0.10	315.1966, 289.1810, 243.1747, 161.0965, 135.0808, 121.0645, 119.0860	Carnosic acid	Terpenes
170	28.59	$[M+H]^+$	$C_{20}H_{30}O_4$	335.2217	0.04	317.2120, 275.2008, 257.1914, 191.1437, 161.0963, 135.0809,	2093050-63-4	Terpenes
171	28.76	$[M+H]^+$	$C_{20}H_{30}O_5$	333.2060	-0.29	315.1961, 187.1109, 159.1169, 121.0648	2093132-39-7	Terpenes
172	28.82	$[M+H]^+$	$C_{20}H_{30}O_3$	319.2268	-0.04	301.2170, 147.1163, 119.0856, 109.1012	83997-21-1	Terpenes
173	28.88	$[M-H]^-$	$C_{42}H_{62}O_{16}$	821.3967	0.19	803.3848, 777.4101, 645.3618, 627.3560, 583.3682, 351.0559, 333.0453, 289.0573, 193.0356, 133.0142, 131.0347, 113.0246, 101.0245	Licoricesaponin H2	Terpenes
174	29.35	$[M+H]^+$	$C_{20}H_{32}O_4$	337.2374	0.19	319.2270, 301.2159, 289.2169, 277.1817, 263.2012, 179.1069, 153.0915, 137.0964, 109.0650, 93.0698	2093050-66-7	Terpenes
175	29.49	$[M+H]^+$	$C_{20}H_{32}O_4$	319.2267	-0.29	301.2166, 273.2221, 161.1320, 119.0856, 109.1015	83997-21-1	Terpenes
176	29.67	$[M+H]^+$	$C_{42}H_{62}O_{16}$	823.4109	-0.47	669.3603, 493.3283, 475.3180, 453.3351, 393.0638, 375.0532, 357.0430, 181.0107, 163.0001, 131.0853, 119.0853, 109.1012	Glycyrrhizic Acid	Terpenes

177	30.14	[M−H]$^-$	$C_{48}H_{76}O_{19}$	955.4910	0.21	497.1145, 339.0920, 321.0841, 113.0246	(3beta,5xi,9xi,22beta)-22,24-Dihydroxyolean-12-en-3-yl 6-deoxy-alpha-L-mannopyranosyl-(1-2)-beta-D-galactopyranuronosyl-(1-2)-beta-D-glucopyranosiduronic acid	Terpenes
178	31.22	[M+H]$^+$	$C_{30}H_{48}O_5$	489.3575	-0.20	435.3276, 407.3333, 219.1748, 201.1639, **189.1638**, 171.1170, 145.1013, 135.1165, 131.0860,	Hovenic acid	Terpenes
179	31.46	[M+H]$^+$	$C_{22}H_{34}O_4$	363.2534	1.26	331.1894, 303.1962, 285.1841, 257.1904, 235.1317, 207.1376, **189.1272**, 161.0961, 133.1017, 121.1016	63399-37-1	Terpenes
180	31.70	[M+H]$^+$	$C_{20}H_{30}O_3$	319.2267	-0.09	283.2052, 255.2109, 241.1958, 191.1794, 161.1321, 149.1322, 135.1168, 121.1012, 107.0855	83997-21-1	Terpenes
181	32.25	[M+H]$^+$	$C_{30}H_{48}O_5$	489.3574	-0.17	435.3245, 407.3309, 389.3226, 219.1744, 201.1638, 191.1795, 161.1325, 149.1325, 135.1173, 107.0855	Esculentic acid	Terpenes
182	32.32	[M+H]$^+$	$C_{20}H_{32}O_3$	321.2424	-0.08	303.2316, 285.2210, 257.2276, 153.1274, 149.1329, 135.1169, 133.1017, 119.0851, 109.1011	3β-Hydroxyanticopalic acid	Terpenes
183	32.44	[M+H]$^+$	$C_{20}H_{30}O_4$	335.2217	-0.10	299.2011, 289.2163, 271.2046, 243.2108, 149.1319, 135.1171	201362-98-3	Terpenes
184	33.15	[M+H]$^+$	$C_{20}H_{32}O_3$	321.2424	-0.09	285.2222, 257.2263, 243.1758, 193.1219, 163.1479, 149.1322, 137.1323, 131.0853, 121.1011	19-Hydroxylabda-8(17),13(Z)-dien-15-oic acid	Terpenes
185	33.15	[M+H]$^+$	$C_{20}H_{28}O$	285.2213	-0.02	267.2123, 257.2247, 133.1015, 119.0854	(9cis)-Retinal	Others

186	33.16	$[M+H]^+$	$C_{20}H_{30}O_2$	303.2318	-0.10	285.2207, 257.2260, 243.2110, 161.1323, 149.1323, 137.1327, 135.1169, 121.1011	Eicosapentaenoic acid	Organic acids
187	33.69	$[M+H]^+$	$C_{20}H_{32}O_2$	305.2475	-0.08	287.2368, 275.2374, 269.2262, 257.2265, 243.2103, 229.1960, 179.1427, 163.1485, 149.1330, 137.0966, 135.1170	Mesterolone	Terpenes
188	33.69	$[M+H]^+$	$C_{20}H_{32}O_2$	305.2475	-0.09	305.2472, 287.2369, 269.2266, 243.2104, 227.1798, 161.1325, 149.1324, 135.1170	Arachidonic acid	Others
189	33.77	$[M+H]^+$	$C_{20}H_{32}O_3$	321.2424	-0.04	285.2204, 163.1485, 151.0747, 137.1326	Agatholic acid	Terpenes
190	33.90	$[M+H]^+$	$C_{20}H_{30}O_3$	319.2267	-0.11	283.2065, 255.2107, 245.1546, 199.1480, 179.1064, 161.1323, 149.1335, 135.1170	(+/-)8-HEPE	Organic acids
191	34.09	$[M+H]^+$	$C_{20}H_{32}O_3$	321.2425	0.08	163.1489, 161.1339, 149.1322, 137.0963, 135.1168, 109.1011	3*β*-Hydroxyanticopalic acid	Terpenes
192	34.12	$[M+H]^+$	$C_{20}H_{28}O_2$	301.2161	-0.32	255.2106, 179.1072, 161.1339, 151.1118, 147.1167, 133.1011, 131.0858, 119.0857	Isotretinoin	Terpenes
193	34.12	$[M+H]^+$	$C_{20}H_{30}O_3$	319.2267	-0.12	283.2075, 255.2107, 193.1237, 179.1068, 161.1332, 151.1111, 133.1014, 121.1012	83997-21-1	Terpenes
194	34.28	$[M+H]^+$	$C_{30}H_{32}O_9$	537.2094	-4.67	415.1745, 397.1668, 386.1684, 373.1284, 371.1487, 357.1339, 356.1269, 341.1407, 339.1229, 325.1072, 313.1072, 311.1296, 297.1128	9-*O*-Feruloyllariciresinol	Lignans

195	34.55	$[M+H]^+$	$C_{20}H_{28}O_2$	301.2162	-0.12	283.2067, 241.1941, 119.0860	Callitrisic acid	Terpenes
196	34.61	$[M+H]^+$	$C_{20}H_{32}O_2$	305.2475	-0.13	287.2368, 269.2262, 243.2107, 227.1804, 187.1490, 149.1327, 133.1005, 121.1011, 109.1012	Mesterolone	Terpenes
197	34.68	$[M+H]^+$	$C_{30}H_{32}O_9$	537.2095	-4.59	415.1755, 371.1484, 351.1240, 343.1156, 297.1122, 283.0971	Lappaol A	Lignans
198	34.87	$[M+H]^+$	$C_{20}H_{30}O$	287.2369	-0.03	269.2271, 119.0856	(+)-Ferruginol	Terpenes
199	35.94	$[M+H]^+$	$C_{30}H_{46}O_4$	471.3467	-0.33	425.3417, 407.3317, 317.2097, 271.2069, 235.1690, 217.1589, 215.1426, 207.1743, 191.1799, **189.1639**, 109.1010	18-*β*-Glycyrrhetinic acid	Terpenes
200	36.00	$[M+H]^+$	$C_{18}H_{30}O_2$	279.2318	0.14	191.1429, 173.1323, 149.0233, 137.1325, 135.1169, 123.1168, 109.1012, 95.0855	*α*-Linolenic acid	Organic acids
201	36.08	$[M+H]^+$	$C_{25}H_{28}O_5$	409.2011	0.25	353.1383, 219.0663, 193.0491, 165.0181	Kushenol A	Lignans
202	36.17	$[M+H]^+$	$C_{18}H_{30}O_2$	279.2318	0.12	261.2217, 191.1429, 163.1122, 151.1489, 149.1324, 137.1324, 135.1169, 123.1168	Pinolenic acid	Organic acids
203	36.49	$[M+H]^+$	$C_{25}H_{26}O_6$	423.1803	0.17	367.1182, 311.0562, 165.0192, 147.0438	Kuwanon C	Terpenes
204	36.78	$[M+H]^+$	$C_{18}H_{30}O_2$	279.2318	0.23	149.0232, 137.1322, 109.1010, 95.0854	*α*-Eleostearic acid	Organic acids
205	36.85	$[M+H]^+$	$C_{30}H_{48}O_4$	473.3624	-0.14	**205.1587**, 189.1662, 107.0855	Corosolic acid	Terpenes
206	37.75	$[M+H]^+$	$C_{21}H_{32}O_4$	349.2374	0.10	317.2118, 303.2338, 299.1995, 243.2103, 193.1225, **189.1633**, 161.1324, 153.0908, 149.1328, 137.1322,	19-Formyloxylabda-8(17),13(E)-dien-15-oic acid	Terpenes
207	37.76	$[M+H]^+$	$C_{20}H_{30}O_4$	335.2217	0.13	317.2115, 121.1012, 109.1013	Agathic acid	Terpenes

208	37.76	$[M+H]^+$	$C_{20}H_{28}O_3$	317.2111	-0.17	299.2001, 289.2176, 271.2058, **189.1639**, 161.1327, 149.1326, 135.1171, 107.0856	18-Hydroxyretinoic acid	Terpenes
209	39.00	$[M+H]^+$	$C_{21}H_{32}O_{22}$	317.2475	-0.06	285.2210, 257.2278, 243.2120, 159.1173, 135.1167, 119.0854	Eicosapentaenoic acid methyl ester	Others
210	39.15	$[M+H]^+$	$C_{30}H_{48}O_4$	473.3626	0.10	437.3422, 427.3572, 409.3457, 391.3369, 357.2803, 331.2629, 315.2331, 301.2166, 207.1752, **205.1594, 189.1631**, 149.1326, 119.0854, 109.1012, 107.0855	Maslinic acid	Terpenes
211	40.31	$[M+H]^+$	$C_{30}H_{46}O_3$	455.3520	-0.10	391.3344, 285.1811, 215.1797, **189.1639**, 175.1474, 161.1328, 145.1015, 133.1015, 119.0855, 109.1012, 107.0857	(3*β*,5ξ,9ξ)-3-Hydroxyoleana-11,13(18)-dien-30-oic acid	Terpenes
212	41.81	$[M+H]^+$	$C_{39}H_{54}O_6$	619.3994	0.05	437.3409, 409.3487, 391.3360, 215.1788, 191.1800, **189.1638**, 165.0548, 163.1487, 147.0440	Rubicoumaric acid	Terpenes
213	43.26	$[M+H]^+$	$C_{30}H_{46}O_2$	439.3569	-0.44	393.3519, 269.2265, **203.1798**, 179.1798, 175.1484, 151.1478, 149.1323, 137.1324, 123.1166, 121.1011, 109.1010, 107.0856	Oleana-2,12-dien-28-oic acid	Terpenes
214#	43.66	$[M+H]^+$	$C_{30}H_{48}O_3$	457.3677	-0.26	393.3519, 249.1854, **205.1949**, 191.1792, **189.1635**, 163.1487, 153.0910, 147.1169, 137.1323, 135.1167, 123.1163, 121.1011, 119.0854, 109.1011, 107.0854	Betulinic acid	Terpenes

215	43.67	$[M+H]^+$	$C_{30}H_{46}O_2$	439.3569	-0.26	393.3537, 249.1860, 215.1800, **205.1956**, 191.1791, **189.1637**, 163.1488, 149.1322, 137.1326, 121.1012, 109.1010	Olean-12-ene-3,11-dione	Terpenes
216	43.94	$[M+H]^+$	$C_{29}H_{46}O$	411.3622	0.08	215.1785, 203.1798, 191.1796, 163.1477, 149.1324, 135.1168, 121.1010, 109.1010	(3*β*,5*α*,20S)-4,4-Dimethylcholesta-8,14,24-trien-3-ol	Terpenes
217#	43.94	$[M+H]^+$	$C_{30}H_{48}O_3$	457.3675	-0.39	393.3521, 249.1850, 191.1795, **189.1636**, 163.1480, 153.0914, 109.1010	Oleanolic acid	Terpenes
219#	44.31	$[M+H]^+$	$C_{30}H_{48}O_3$	457.3676	-0.39	269.8506, 215.1789, 203.1809, 179.1798, 161.1328, 137.1330, 131.0862, 121.1013, 109.1009	Ursolic acid	Terpenes
219	44.36	$[M+H]^+$	$C_{30}H_{48}O$	425.3778	-0.04	407.3679, 163.1478, 257.2270, 191.1801, 161.1330, 217.1948, 149.1323, 137.1323, 109.1011	*β*-Amyrone	Terpenes
220	46.02	$[M+H]^+$	$C_{30}H_{48}O$	425.3778	-0.04	191.1803, 163.1482, 161.1330, 149.1325, 137.1320, 131.0859, 123.1174, 109.1009	δ-Amyrone	Terpenes
221	46.21	$[M+H]^+$	$C_{32}H_{50}O_2$	467.3882	0.26	407.3662, 217.1947, **203.1793**, 191.1794, 163.1491, 161.1324, 149.1321, 135.1166, 121.1010, 109.1011	Lupa-13(18),20(29)-dien-3-yl acetate	Terpenes
222	46.79	$[M+H]^+$	$C_{30}H_{48}O$	425.3778	-0.04	407.3662, 217.1950, **203.1789**, 191.1793, 163.1481, 161.1326, 149.1325, 137.1324, 123.1168, 109.1011	*β*-Amyrone	Terpenes
223	47.18	$[M+H]^+$	$C_{24}H_{38}O$	343.2996	0.09	325.2893, 191.1816, 147.1180, 137.1331, 135.1171, 109.1014, 107.0859	(22E)-Chola-5,22-dien-3-ol	Terpenes

224	47.41	$[M+H]^+$	$C_{32}H_{50}O_2$	467.3882	-0.31	407.3680, 203.1793, 191.1793, **189.1642**, 163.1483, 161.1325, 149.1327, 135.1167, 131.0855, 109.1011	Lupa-13(18),20(29)-dien-3-yl acetate	Terpenes
225*	48.05	$[M+H]^+$	$C_{34}H_{40}O_9$	593.2759	2.39	565.2780, 564.2382, 561.2492, 533.2545, 505.2206, 473.2394, 461.2311, 447.2159, 445.1995, 431.1816	Unknown	Terpenes
226	49.33	$[M+H]^+$	$C_{30}H_{50}O$	427.3933	-0.24	409.3828, 219.2109, **205.1955**, 191.1797, 161.1326, 151.1482, 149.1326, 135.1168, 121.1010	Lupeol	Terpenes
227	51.08	$[M+H]^+$	$C_{30}H_{50}O$	427.3934	0.05	409.3840, 123.1177, 109.1014, 93.0701	ψ-Taraxasterol	Terpenes
228	54.29	$[M+H]^+$	$C_{30}H_{48}O$	425.3778	-0.01	425.3791, 257.2280, 217.1950, **203.1793**, 191.1792, 163.1487, 149.1323, 137.1318, 135.1166, 119.0855, 109.1011, 107.0854, 95.0855	*β*-Amyrone	Terpenes
229	55.06	$[M+H]^+$	$C_{32}H_{54}O_4$	503.4090	0.22	425.3754, 217.1944, 191.1792, 163.1479, 161.1326, 149.1327, 135.1169, 109.1011	Ocotillol monoacetate	Terpenes
230	56.09	$[M+H]^+$	$C_{32}H_{52}O_2$	469.4038	-0.47	409.3846, 219.2123, 191.1794, 163.1480, 149.1324, 137.1323, 123.1170, 109.1011, 107.0854, 95.0855	Taraxasteryl acetate	Terpenes
231	56.86	$[M+H]^+$	$C_{30}H_{50}O$	427.3937	0.01	409.3822, 353.3213, 191.1800, 149.1323, 137.1326, 109.1011, 97.1013	ψ-Taraxasterol	Terpenes
232	56.86	$[M+H]^+$	$C_{32}H_{52}O_2$	469.4039	-0.31	409.3831, 353.3225, 257.2261, 229.1951, 217.1953, 191.1795, 163.1485, 149.1323, 137.1330, 123.1168, 109.1013	*β*-Amyrin acetate	Terpenes

233	56.86	$[M+H]^+$	$C_{32}H_{54}O_3$	487.4136	-0.32	409.3837, 353.3210, 327.3050, 299.2734, 229.1952, 199.1486, 149.1328, 135.1170, 123.1167, 109.1013	Dammar-24-ene-3,20-diol, 3-acetate, (3*β*)-	Terpenes
234	56.97	$[M+H]^+$	$C_{32}H_{50}O_3$	483.3832	-0.08	423.3634, 277.1796, **189.1639**, 149.1332, 133.1011, 123.1173, 109.1011, 105.0698	11-Oxours-12-en-3-yl acetate	Terpenes
235	57.11	$[M+H]^+$	$C_{30}H_{52}O_2$	444.4043	0.77	149.1337, 135.1167, 123.1171, 109.1014	(3*β*,8*α*,9*β*)-Lanost-24-ene-3,20-diol	Terpenes
236	57.74	$[M+H]^+$	$C_{32}H_{52}O_2$	469.4040	-0.06	409.3834, 241.1938, 229.1948, 215.1797, 187.1482, 163.1491, 161.1322, 149.1319, 135.1173, 133.1015, 131.0857, 123.1173, 119.0856, 109.1010, 107.0859, 95.0855, 81.0699	Isobauerenyl acetate	Terpenes
237	58.65	$[M+Na]^+$	$C_{32}H_{50}O_2$	467.3883	-0.22	407.3681, 243.2109, **203.1805**, 191.1797, 163.1486, 149.1326, 137.1325, 135.1166, 131.0852, 121.1015, 109.1012	Lupa-13(18),20(29)-dien-3-yl acetate	Terpenes

Note: #identified by comparison with reference standards, * potential novel compound. PhGs-Phenylethanoid glycosides

Table S2. The detail information of all 29 reference standards used in this study.

	NO.	Compounds	Molecular Formula	Molecular Weight	CAS Number	Class
LC-MS	1	Forsythoside A	$C_{29}H_{36}O_{15}$	624.2054	79916-77-1	Phenylethanoid glycosides
	2	Forsythoside I	$C_{29}H_{36}O_{15}$	624.2054	1177581-50-8	
	3	Isoforsythiaside	$C_{29}H_{36}O_{15}$	624.2054	1357910-26-9	
	4	Acteoside	$C_{29}H_{36}O_{15}$	624.2054	61276-17-3	
	5	Isoacteoside	$C_{29}H_{36}O_{15}$	624.2054	61303-13-7	
	6	Forsythoside B	$C_{34}H_{44}O_{19}$	756.2477	81525-13-5	
	7	Forsythoside E	$C_{20}H_{30}O_{12}$	462.1737	93675-88-8	
	8	Calceolarioside B	$C_{23}H_{26}O_{11}$	478.1475	105471-98-5	
	9	Sallidroside	$C_{29}H_{36}O_{15}$	300.1209	61276-17-3	
	10	Phillygenin	$C_{21}H_{24}O_6$	372.1573	487-39-8	Lignans
	11	Phillyrin	$C_{27}H_{34}O_{11}$	534.2101	487-41-2	
	12	(+)-Pinoresinol-4′-*O*-*β*-D-glucopyranoside	$C_{26}H_{32}O_{11}$	520.1945	69251-96-3	
	13	Arctiin	$C_{27}H_{34}O_{11}$	534.2101	20362-31-6	
	14	Rutin	$C_{27}H_{30}O_{16}$	610.1534	153-18-4	Flavonoids
	15	Cornoside	$C_{14}H_{20}O_8$	316.1158	40661-45-8	Cyclohexyl ethanol derivatives
	16	Forsythenside B	$C_{22}H_{26}O_{11}$	466.1475	202721-10-6	
	17	Adoxosidic acid	$C_{16}H_{24}O_{10}$	510.1737	84375-46-2	Iridoids
	18	Ursolic acid	$C_{30}H_{48}O_3$	456.3604	77-52-1	Terpenoids
	19	Oleanolic acid	$C_{30}H_{48}O_3$	456.3604	508-02-1	
	20	Corosolic acid	$C_{30}H_{48}O_4$	472.3553	4547-24-4	
	21	Betulinic acid	$C_{30}H_{48}O_3$	456.3604	472-15-1	
HS-GC-MS	1	*α*-pinene	$C_{10}H_{16}$	136.1252	80-56-8	Terpenes
	2	*β*-pinene	$C_{10}H_{16}$	136.1252	127-91-3	
	3	*α*-terpinene	$C_{10}H_{16}$	136.1252	99-86-5	
	4	Camphene	$C_{10}H_{16}$	136.1252	79-92-5	
	5	*γ*-terpinene	$C_{10}H_{16}$	136.1252	99-85-4	
	6	(+)-*δ*-cadinene	$C_{15}H_{24}$	204.1878	483-76-1	
	7	*α*-terpineol	$C_{10}H_{18}O$	154.1358	98-55-5	Alcohols
	8	Terpinen-4-ol	$C_{10}H_{18}O$	154.1358	562-74-3	

Table S3. The detailed information of Forsythiae Fructus samples.

NO	Sample Code	Sources	Collection date	NO	Sample Code	Sources	Collection date
1	GFF-1	Xinxiang, Henan	July 2022	21	RFF-1	Luoyang, Henan	August 2022
2	GFF-2	Jiaozuo, Henan	July 2022	22	RFF-2	Xinxiang, Henan	August 2022
3	GFF-3	Luoyang, Henan	July 2022	23	RFF-3	Jiaozuo, Henan	August 2022
4	GFF-4	Changzhi, Shanxi	July 2022	24	RFF-4	Xinxiang, Henan	August 2022
5	GFF-5	Lvliang, Shanxi	July 2022	25	RFF-5	Jiaozuo, Henan	August 2022
6	GFF-6	Jinzhong, Shanxi	July 2022	26	RFF-6	Luoyang, Henan	August 2022
7	GFF-7	Yuncheng, Shanxi	July 2022	27	RFF-7	Changzhi, Shanxi	August 2022
8	GFF-8	Cangzhou, Hebei	July 2022	28	RFF-8	Lvliang, Shanxi	August 2022
9	GFF-9	Zhangjiakou, Henan	July 2022	29	RFF-9	Jinzhong, Shanxi	August 2022
10	GFF-10	Linyi, Shangdong	July 2022	30	RFF-10	Yuncheng, Shanxi	August 2022
11	GFF-11	Huangshan, Anhui	July 2022	31	RFF-11	Cangzhou, Hebei	August 2022
12	GFF-12	Jincheng, Shanxi	July 2022	32	RFF-12	Zhangjiakou, Henan	September 2022
13	GFF-13	Jincheng, Shanxi	July 2022	33	RFF-13	Linyi, Shangdong	September 2022
14	GFF-14	Jinzhong, Shanxi	July 2022	34	RFF-14	Huangshan, Anhui	September 2022
15	GFF-15	Yuncheng, Shanxi	July 2022	35	RFF-15	Jincheng, Shanxi	September 2022
16	GFF-16	Changzhi, Shanxi	July 2022	36	RFF-16	Yuncheng, Shanxi	September 2022
17	GFF-17	Lvliang, Shanxi	July 2022	37	RFF-17	Ningchuan, Shaanxi	September 2022
18	GFF-18	Luoyang, Henan	July 2022	38	RFF-18	Yuncheng, Shanxi	September 2022
19	GFF-19	Xinxiang, Henan	July 2022	39	RFF-19	Danfeng, Shaanxi	September 2022
20	GFF-20	Jiaozuo, Henan	July 2022	40	RFF-20	Danfeng, Shaanxi	September 2022
				41	RFF-21	Yuncheng, Shanxi	September 2022
				42	RFF-22	Yuncheng, Shanxi	September 2022
				43	RFF-23	Luoyang, Henan	September 2022

	44	RFF-24	Xinxiang, Henan	September 2022
	45	RFF-25	Xinxiang, Henan	October 2022
	46	RFF-26	Yuncheng, Shanxi	October 2022
	47	RFF-27	Jincheng, Shanxi	October 2022
	48	RFF-28	Jiaozuo, Henan	October 2022
	49	RFF-29	Huangshan, Anhui	October 2022
	50	RFF-30	Yuncheng, Shanxi	October 2022

Table S4. Differential compounds identified through LC-MS and HS-GC-MS analysis.

Analytical technique	NO	Differential compounds	VIP Score	*p*-value	FC (GFF/RFF)	Class
LC-MS	L1	Quinic acid	1.24	3.77E-09	4.29	Organic acids
	L2	Hydroxytyrosol 1-*O*-glucoside	1.52	1.55E-09	9.06	Cyclohexyl ethanol derivatives
	L3	Cornoside	1.52	4.98E-10	7.67	Cyclohexyl ethanol derivatives
	L4	Rengynic acid-1-*O*-*β*-D-glucopyranoside	1.24	1.22E-13	4.47	Cyclohexyl ethanol derivatives
	L5	Vanilloloside	1.4	3.87E-08	6.92	Organic acids
	L6	Forsythoside D	1.43	3.58E-08	7.11	PhGs
	L7	Adoxosidic acid	1.28	1.09E-08	4.47	Iridoids
	L8	Forsythide	1.23	1.86E-09	5.82	Iridoids
	L9	Salidroside	1.35	2.65E-09	6.77	PhGs
	L10	Rebouoside B	1.11	5.11E-07	4.50	PhGs
	L11	Forsythoside E	1.02	2.35E-06	3.61	PhGs
	L12	Darendoside A	1.26	1.34E-10	3.92	PhGs
	L13	Forsythenside B	1.24	1.93E-13	5.54	Cyclohexyl ethanol derivatives
	L14	4-*O*-*p*-Coumaroylquinic acid	1.1	7.00E-06	2.73	Organic acids
	L15	Rengyoside D	1.13	4.41E-12	3.71	Cyclohexyl ethanol derivatives
	L16	Hastatoside	1.19	3.42E-05	6.06	Iridoids
	L17	Calceolarioside A	1.02	2.54E-13	2.99	PhGs
	L18	(+)-8-Hydroxypinoresinol 4-*O*-*β*-D-glucopyranoside	1.2	1.76E-13	3.92	Lignans
	L19	Forsythiayanoside B	1.44	7.56E-11	6.54	Lignans
	L20	Forsythialanside E	1.19	3.66E-14	3.86	Lignans
	L21	Calceolarioside C	1.28	4.02E-09	6.02	PhGs
	L22	Isoforsythiaside	1.1	8.34E-06	2.95	PhGs
	L23	S-Suspensaside methyl ether	1.54	2.44E-16	0.10	Lignans
	L24	Plantainoside A	1.3	3.09E-15	4.38	PhGs
	L25	Adoxosidic acid-6’-oleuroperic ester	1.17	2.64E-12	3.39	Iridoids
	L26	Forsythoside A	1.19	7.44E-13	3.58	PhGs
	L27	Acteoside	1.05	1.16E-14	2.68	PhGs
	L28	Calceolarioside B	1.35	8.17E-11	6.50	PhGs
	L29	Kaempferol 3-*O*-rutinoside	1.02	5.83E-12	2.62	Flavonoids
	L30	Forsythenside L	1.57	6.51E-13	6.59	Cyclohexyl ethanol derivatives
	L31	(+)-Pinoresinol 4′-*O*-*β*-D-glucopyranoside	1.39	1.37E-13	6.50	Lignans
	L32	Forsyshiyanine B	1.09	6.30E-04	0.18	Alkaloids
	L33	Isoacteoside	1.09	1.79E-09	3.03	PhGs
	L34	Fraxiresinol-4′-*O*-*β*-D-glucopyranoside	1.26	3.12E-11	5.10	Lignans

	L35	Matairesinoside	1.06	7.61E-09	3.36	Lignans
	L36	Forsydoitriside A	1.08	1.85E-08	3.81	Iridoids
	L37	Suspenoidside A	1.19	3.10E-09	3.81	Iridoids
	L38	Acanthoside B	1.33	3.70E-12	5.03	Lignans
	L39	Suspenoidside E	1.24	5.13E-12	3.32	Iridoids
	L40	Suspenoidsides D	1.35	3.47E-12	4.86	Iridoids
	L41	Esculentic acid	1.19	6.90E-09	0.32	Terpenes
	L42	18-β-Glycyrrhetinic acid	1.04	2.09E-19	0.42	Terpenes
	L43	Maslinic acid	1.09	2.75E-17	0.36	Terpenes
HS-GC-MS	H1	Cyclene	1.26	3.01E-02	1.30	Terpenes
	H2	α-thujene	1.28	6.88E-03	1.18	Terpenes
	H3	α-Pinene	1.38	1.24E-16	1.92	Terpenes
	H4	Camphene	1.36	2.26E-17	1.99	Terpenes
	H5	Sabinene	1.33	6.86E-10	1.84	Terpenes
	H6	β-Pinene	1.36	4.85E-14	1.71	Ketones
	H7	6-Methylhept-5-en-2-one	1.05	3.37E-10	0.56	Terpenes
	H8	α-Phellandrene	1.25	1.41E-02	0.75	Aromatic
	H9	o-Cymene	1.28	1.02E-02	0.80	Terpenes
	H10	(R)-Isocarvestrene	1.32	2.56E-05	1.21	Alcohols
	H11	Eucalyptol	1.29	1.44E-04	1.21	Aldehydes
	H12	Phenylacetaldehyde	1.12	1.30E-13	0.72	Terpenes
	H13	Terpinolene	1.22	5.05E-05	0.71	Alcohols
	H14	Linalool	1.10	4.58E-04	0.59	Ketones
	H15	Thujone	1.22	7.97E-03	0.68	Esters
	H16	4-Terpinenyl acetate	1.01	1.17E-10	0.52	Ketones
	H17	Sabinone	1.30	1.14E-02	0.73	Aldehydes
	H18	Cuminaldehyde	1.10	1.65E-02	0.73	Terpenes

Table S5. Performance and validation results of OPLS-DA models for GFF and RFF classification based on single technique and mid-level data fusion.

**Model parameters**	**LC-MS**		**HS-GC-MS**	**Mid-level data fusion**
	**ESI⁺**	**ESI⁻**		
No. variables	798	1071	67	43+18
R^2X	0.806	0.836	0.825	0.891
R^2Y	0.994	0.993	0.968	0.986
Q^2	0.976	0.979	0.930	0.974
NC[a]	1+3	1+3	1+3	1+3
Permutation test (R^2; Q^2)	0.541; -0.637 (GFF)<br>0.536; -0.651 (RFF)	0.556; -0.554 (GFF)<br>0.552; -0.598 (RFF)	0.382; -0.564 (GFF)<br>0.386; -0.526 (RFF)	0.340; -0.604 (GFF)<br>0.341; -0.589 (RFF)
CV-ANOVA *p*-value	1.14444e-030	8.29891e-029	1.45136e-021	3.15998e-025

a NC: No. of components indicated as "No. of predictive components + No. of orthogonal components"

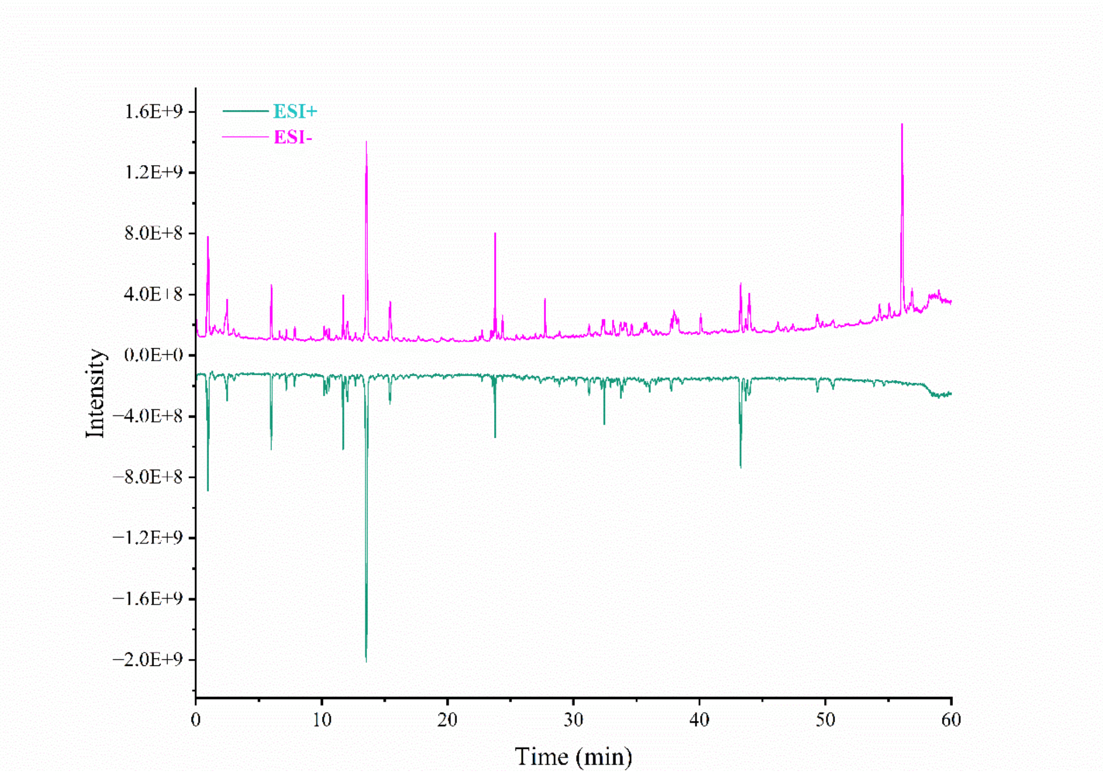

Figure S1. Total ion chromatograms (TICs) for the QC sample in positive ion modes (ESI^{+}) and negative ion modes (ESI^{-}).

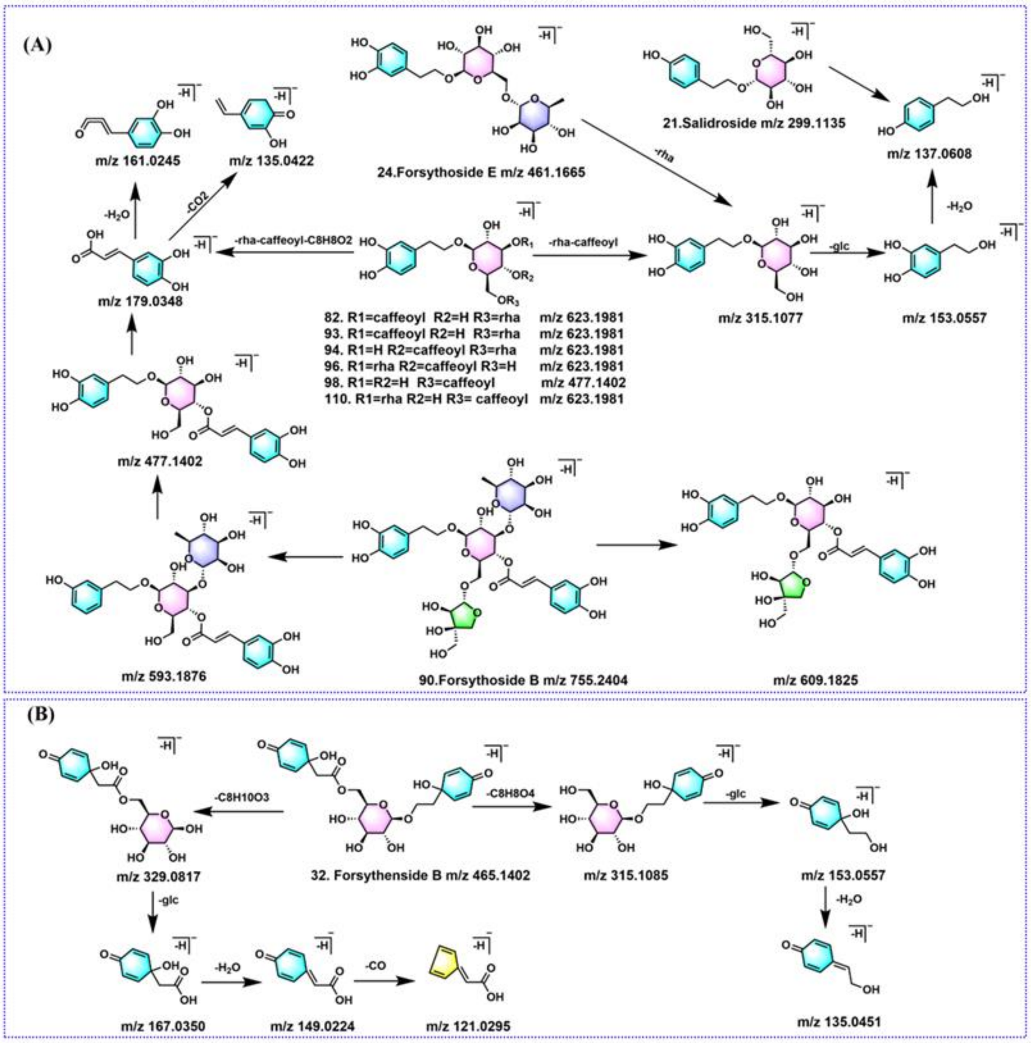

Figure S2. The fragmentation behavior of (A) phenylethanoid glycosides and (B) Forsythenside B in negative ion mode.

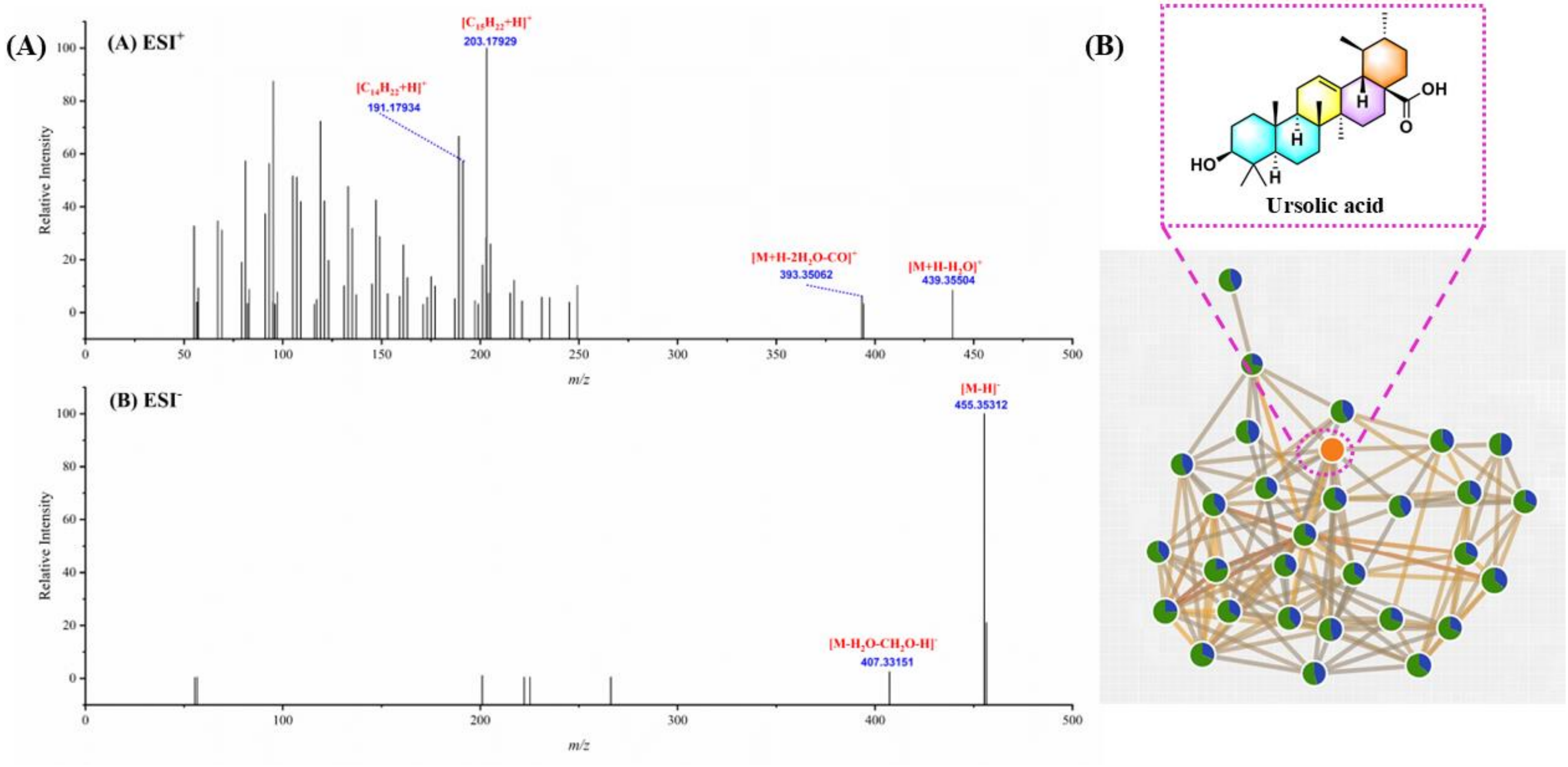

Figure S3. The MS/MS spectra of ursolic acid in ESI$^+$ and ESI$^-$ (A). Molecular network analysis with CD software identified 30 triterpenoid compounds in ESI$^+$ mode.

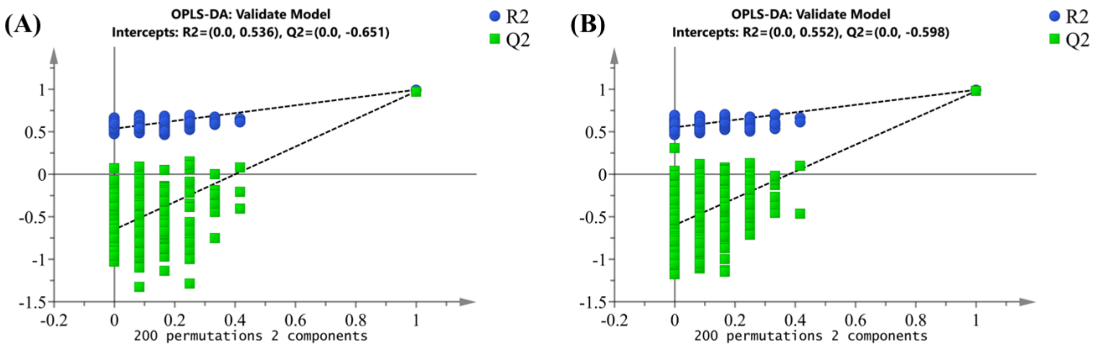

Figure S4. 200 times permutation test plots: (A) ESI^+ mode with 798 features and (B) ESI^- mode with 1,071 features.

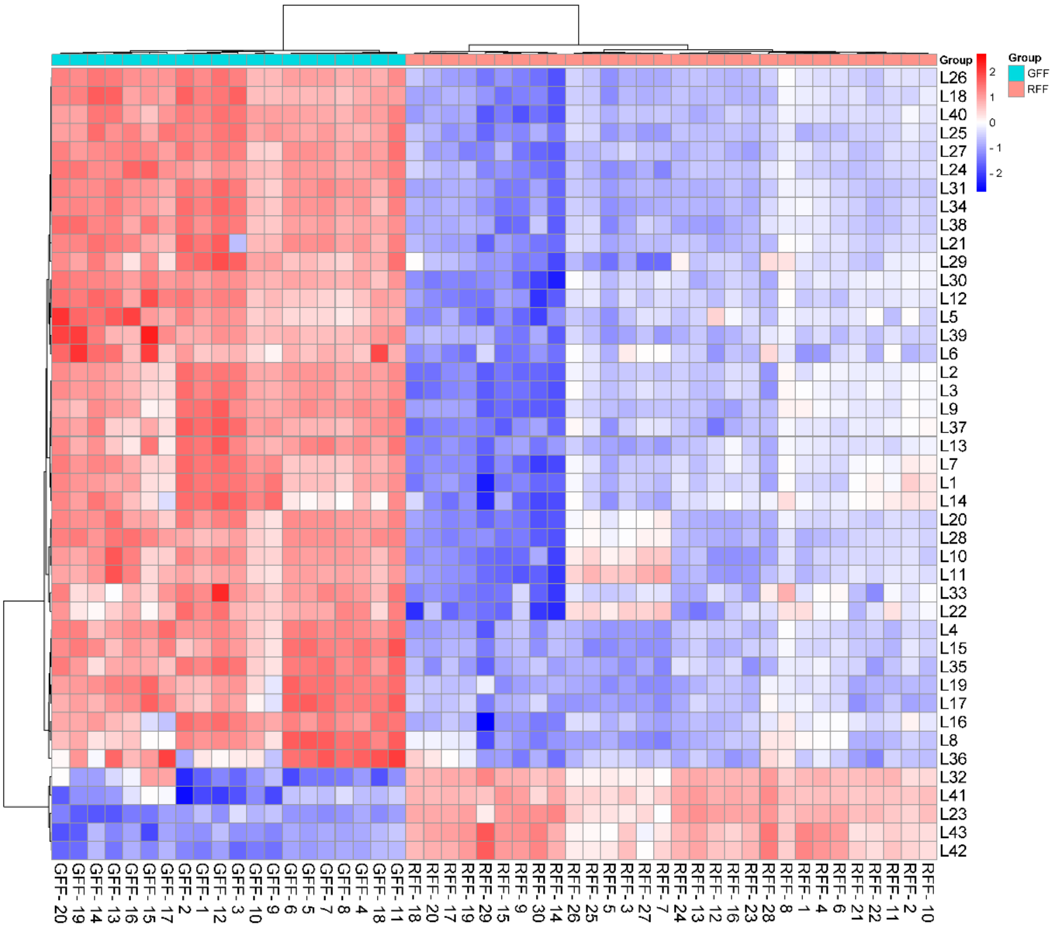

Figure S5. Heatmap of 43 differential non-volatile compounds.

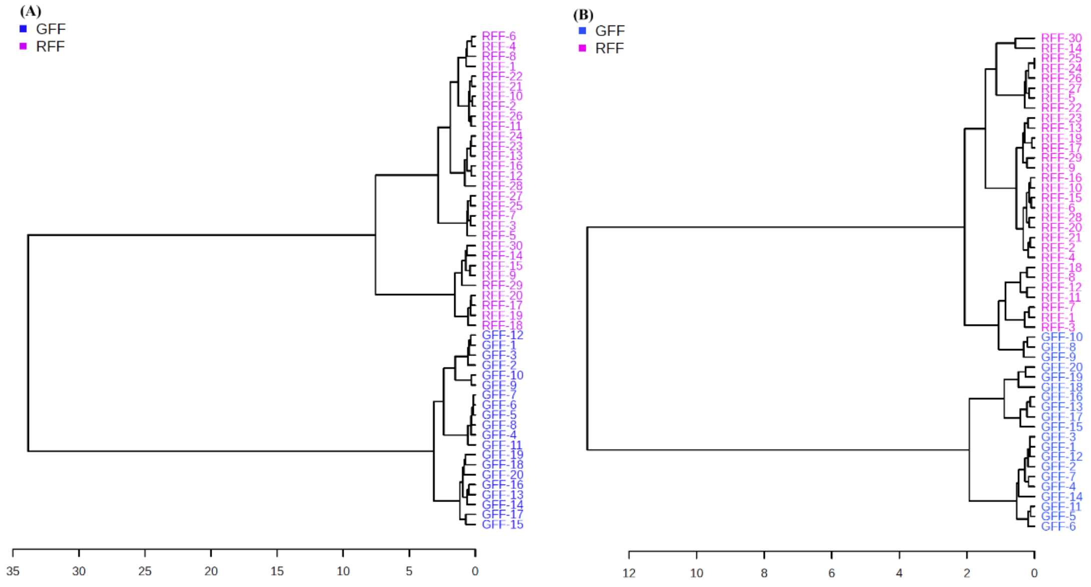

Figure S6. Hierarchical Clustering Analysis (HCA) based on the differential metabolites screened by LC-MS (A) and HS-GC-MS (B).
